# Markedly Improved Catalytic Dehydration of Sorbitol to Isosorbide by Sol–Gel Sulfated Zirconia: A Quantitative Structure–Reactivity Study

**DOI:** 10.1021/acscatal.3c00755

**Published:** 2023-07-19

**Authors:** Jack T. Hopper, Ruining Ma, James B. Rawlings, Peter C. Ford, Mahdi M. Abu-Omar

**Affiliations:** † Department of Chemistry and Biochemistry, University of California Santa Barbara, Santa Barbara, California 93106, United States; ‡ Department of Chemical Engineering, University of California Santa Barbara, Santa Barbara, California 93106, United States

**Keywords:** sulfated zirconia, sol−gel synthesis, acid catalyst, kinetic modeling, sorbitol, isosorbide, biomass conversion

## Abstract

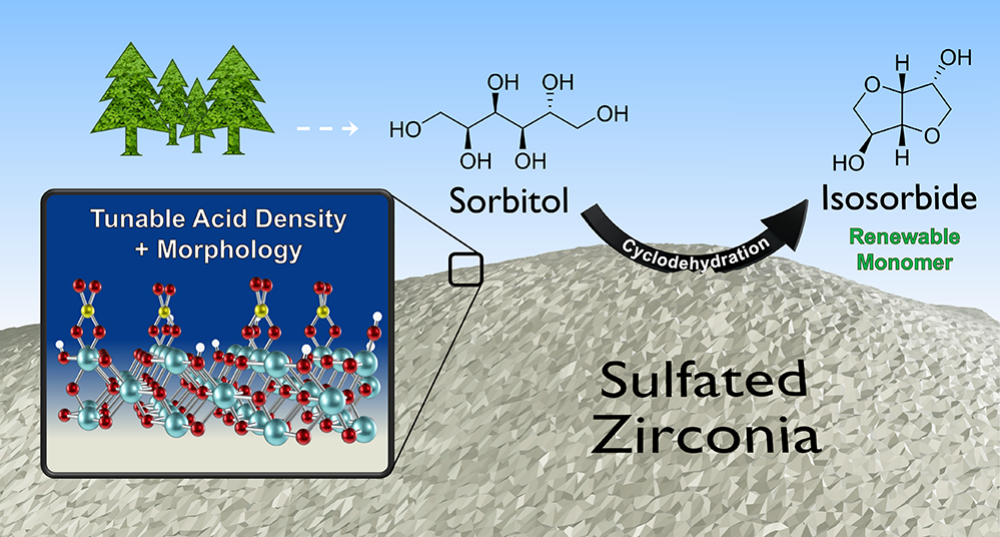

Isosorbide, a bicyclic C6 diol, has considerable value as a precursor for the production of bio-derived polymers. Current production of isosorbide from sorbitol utilizes homogeneous acid, commonly H_2_SO_4_, creating harmful waste and complicating separation. Thus, a heterogeneous acid catalyst capable of producing isosorbide from sorbitol in high yield under mild conditions would be desirable. Reported here is a quantitative investigation of the liquid-phase dehydration of neat sorbitol over sulfated zirconia (SZ) solid acid catalysts produced via sol–gel synthesis. The catalyst preparation allows for precise surface area control (morphology) and tunable catalytic activity. The S/Zr ratio (0.1–2.0) and calcination temperature (425–625 °C) were varied to evaluate their effects on morphology, acidity, and reaction kinetics and, as a result, to optimize the catalytic system for this transformation. With the optimal SZ catalyst, complete conversion of sorbitol occurred in <2 h under mild conditions to give isosorbide in 76% yield. Overall, the quantitative kinetics and structure–reactivity studies provided valuable insights into the parameters that govern product yields and SZ catalyst activity, central among these being the relative proportion of acid site types and Brønsted surface density.

## Introduction

The shift toward renewable fuels and chemicals is an increasingly important transition facing society. With a low-cost, global ubiquity, and abundant supply, cellulosic biomass represents a sustainable alternative to petroleum-based feedstocks.^1−3^ However, biomass derivatives possess fundamentally different chemical compositions to petroleum, and therefore it has become necessary to develop new methods for their transformation. With a relatively high oxygen content,^4^ biomass frequently requires catalytic deoxygenation to increase C/O closer to those found in traditional commodity chemicals. Reactions such as dehydration,^5^ hydrogenolysis,^6^ and deoxydehydration^7^ have been successfully employed for this purpose.

Often, the goal of these biomass transformations is to synthesize common drop-in chemicals such as olefins, alcohols, and aromatics more sustainably.^3^ An alternative approach is to take advantage of functional groups already present to produce novel platform chemicals for the next generation of materials and products. To these ends, the US Department of Energy (DOE) established a preliminary list in 2004,^8^ and subsequently updated in 2010,^9^ of the top biomass-derived molecules for their promise in large-scale utilization. Among them, sorbitol was one of the highest scoring renewable platform chemicals across all criteria owing to its ubiquity, favorable economics, and potential for a wide variety of transformations.

Readily produced in high yield via hydrogenation of glucose (derived from cellulose), sorbitol is a low-cost ($0.65/kg) C6 sugar alcohol widely used in the food, pharmaceutical, and cosmetic industries. Its current production therefore exceeds 500,000 tons per year.^10,11^ Successive cyclodehydrations yields isosorbide, a diol used to make products such as polyethers,^12^ polyesters,^12,13^ flame retardants,^14^ and environmentally benign high-boiling solvents.^15^ Polymers produced from isosorbide are rigid, non-toxic, and often biodegradable.^16^ Isosorbide has also been used as an additive to polyethylene terephthalate (PET), improving the polymer’s strength and glass transition temperature (*T*_g_).^17^

Current production of isosorbide employs sulfuric acid (H_2_SO_4_);^18^ however, this homogeneous acid prompts costly, complex separations, produces harmful waste, and can require extended reaction times.^19^ Solid acid catalysts have therefore been investigated for production of isosorbide from sorbitol and include zeolites, acid resins, acidic polymers, and supported ionic liquids.^20−26^ Among them are a SiO_2_-Al_2_O_3_ catalyst studied by Li and Huber that achieved an isosorbide yield of 62% at 245 °C under 29 bar He after 24 h,^27^ and BPO_4_ studied by Rusu et al., producing isosorbide with 76% selectivity in 24 h at 220 °C.^28^ Despite respectable isosorbide production, these systems are limited by high catalyst cost, poor thermal stabilities, and/or require high temperatures and long reaction times.

The sulfated metal oxides, on the other hand, are known for their excellent thermal stabilities and high acidities, with several studied as catalysts for sorbitol dehydration.^29−33^ Among them, sulfated zirconia (SZ) is particularly attractive because of its low cost, non-toxicity, and large-scale availability.^34^ In the decades since the pioneering work by Hino et al. in 1979,^35^ a wealth of research has been reported on the use of SZ in a variety of industrially relevant reactions ranging from alkane isomerization^36−38^ and alkylation,^39^ to biomass transformations including glucose conversion^40^ and sorbitol dehydration. For example, upon complete sorbitol conversion over SZ, Khan et al. obtained isosorbide in 62% yield in 2 h at 210 °C;^41^ while more recently, Zhang et al. achieved 29% yield at 180 °C after 2 h.^42^ Although possessing high activities, SZ and other sulfated oxides frequently necessitate elevated temperatures that increase formation of oligomeric byproducts, thereby lowering product yields.^43^ Additionally, reduced pressure is commonly used to accelerate dehydration,^31−33,41,44,45^ which, along with elevated reaction temperatures, results in energy-intensive processes: key motivators in this study.

**Scheme 1:**
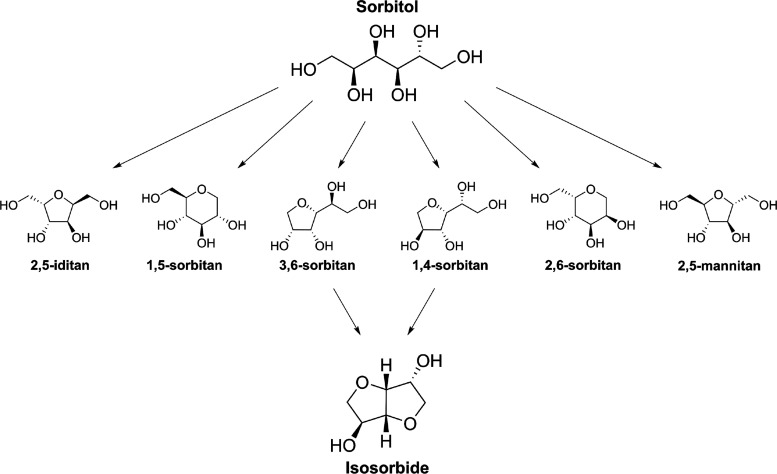
Cyclodehydration of Sorbitol to Isosorbide Can Give Various Anhydrohexitol Intermediates, of Which Only Two Proceed Successfully to Afford Isosorbide

In SZ syntheses, the choice of Zr precursor (typically Zr(OH)_4_) has a marked effect on the final catalytic properties.^29^ Classic wetness impregnation of Zr(OH)_4_ however does not provide control of the resulting particles and their morphological properties. In this study, we utilize a sol–gel method as an alternate synthetic pathway to SZ, enabling increased command over these important parameters.^46^ Such control is particularly valuable because it affects the prevailing intermediates (Scheme 1), and as a result, the overall reaction selectivity towards isosorbide.^45^ Notably, the use of sol–gel prepared SZ has not previously been reported for sorbitol dehydration. Herein, we describe the effects of systematic variation in the S/Zr ratio and calcination temperature on catalyst acidity and surface area as descriptors controlling activity and selectivity. A neat (solventless) reaction under ambient pressure affords isosorbide in 76% selectivity at moderate reaction temperature (150 °C) in less than 2 h. Isosorbide is isolated with a simple solid–liquid separation step. In addition, investigation of the postreaction catalyst led to devising an effective regeneration methodology, enabling SZ to be recycled at least five times. Detailed kinetic analysis and modeling combine to provide a plausible mechanism.

## Experimental Details

### Catalyst Preparation

A variety of SO_4_/ZrO_2_ (SZ) catalysts were synthesized with varying S/Zr molar ratios (0.1–2) via a modified sol–gel synthesis using zirconium *n*-propoxide (Zr(OC_3_H_7_)_4_) (Thermo Fisher Scientific, 70 wt % in *n*-propanol). The starting 70 wt % zirconium *n*-propoxide solution was diluted to 12.5 wt % with dry *n*-propanol under Ar and added dropwise to a quantity of *aq.* H_2_SO_4_ spanning 0.1–2.0 M with vigorous stirring. The resulting white slurry was allowed to stir under ambient conditions for 4 h and then dehydrated overnight at 120 °C. The obtained solid was ground to a fine powder and calcined under flowing air (50 sccm/4 h) to generate the final SZ catalyst. Catalysts were stored in air and used without any further treatment. The label “*x*-SZ-*y”* was used to designate specific catalysts in which *x* corresponds to the S/Zr ratio and *y* denotes the calcination temperature (425, 525, or 625 °C). If the temperature is not specified, 625 °C calcination was the default condition. A non-sulfated (NS) sol–gel ZrO_2_ sample was prepared in the manner described above using deionized water in place of H_2_SO_4_.

### Catalyst Characterization

The surface areas, pore volumes, and average pore diameters were determined via N_2_ physisorption at 77 K using a Micromeritics 3Flex porosimeter. Prior to analysis, samples were degassed at 150 °C for 2 h under vacuum to remove any physically adsorbed water. Surface area was calculated by the Brunauer–Emmett–Teller (BET) method in the range of P/P_0_ = 0.05–0.3 where a linear correlation coefficient of 0.999 was exceeded and a positive BET C value was established in the BET transform plot.^47^ Pore volumes and diameters were determined by applying the Barrett–Joyner–Halenda (BJH) model to the isotherm desorption branch.^48^ Micromeritics Flex Version 6.01 software was used for data analysis.

Infrared spectra of catalysts were obtained with a Thermo Scientific Nicolet iS10 Fourier transform infrared (FTIR) spectrometer with a diamond Attenuated Total Reflection (ATR) probe to determine the identity of specific surface species and coordination modes. Catalysts were imaged in air without pretreatment to ascertain relevant species present. Separate infrared spectra of spent and regenerated catalysts were recorded via diffuse reflectance infrared Fourier transform spectroscopy (DRIFTS) with a Thermo Scientific Praying Mantis Diffuse Reflectance Accessory. Spectra were obtained in the range of 500–4000 cm^–1^.

Scanning electron microscopy (SEM) images were acquired on a FEI Nova Nano 650 FEG microscope equipped with a high stability Schottky field emission gun and large specimen chamber. The catalyst samples were loaded to double-sided copper foil tape with conductive adhesive, which allows the catalyst sample to be stabilized on the SEM aluminum stub. Prior to imaging, the SEM chamber was evacuated to 8 × 10^–5^ mbar. The catalyst image was taken by Everhart–Thornley detector (ETD) with the beam voltage between 5 and 10 keV for high-resolution secondary election (SE mode) SEM imaging.

Thermogravimetric analysis (TGA) of fresh, spent, and regenerated catalysts was accomplished on a Discovery TGA 5500 instrument to evaluate the thermal stability and mass loss with temperature. The sample was placed in Al_2_O_3_ cup suspended on a Pt hanging pan and heated at a rate of 10 °C/min from 35 to 1000 °C under air.

Ammonia temperature-programmed desorption (NH_3_-TPD) was performed using an Autochem II 2920 chemisorption analyzer for determination of acid site loading. Prior to analysis, the sample was dehydrated at 150 °C after ramping at 10 °C/min under He (UHP, Airgas) flow for 1 h. The sample was then cooled to 30 °C and exposed to 10% NH_3_/He (Airgas) for 0.5 h. Following NH_3_ exposure, the sample was analyzed for desorption behavior from 30 to 700 °C, heating at a rate of 10 °C/min. The total number of acid sites AS_TPD_ (Brønsted and Lewis) obtained from the NH_3_-TPD experiments was used to calculate the TOF_isosorbide_, using eq 1,
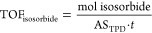
1where AS_TPD_ is the mass-normalized number of catalyst acid sites and *t* is the reaction time.

To calculate the relative proportions of Brønsted and Lewis acid types, the infrared spectra of coordinated pyridine were recorded using a Thermo Scientific Nicolet iS10 FTIR spectrometer. Typically, samples were loaded into a Harrick Scientific High Temperature Reaction Chamber and placed within a Thermo Scientific Praying Mantis Diffuse Reflectance Accessory. Dehydration then proceeded under Ar at 150 °C for 1 h following a ramping rate of 10 °C/min. After the allotted time, samples were cooled to 30 °C and background scans were collected prior to pyridine exposure. Ar was then bubbled through pyridine and into the reaction chamber for 0.5 h to saturate the catalyst surface followed by Ar flush for 0.5 h. Pyridine coordination to Brønsted (Py-H^+^) and to Lewis acid sites (Py-L) was assigned to infrared absorbances located at 1530–1540 and 1445 cm^–1^, respectively.^49^ Following baseline correction, the relevant peaks were integrated and normalized using corresponding molar extinction coefficients, ε(Py-H^+^) or ε(Py-L).^50,51^ The obtained normalized peak areas were then used to calculate B/L, the proportion of Brønsted to Lewis acid sites. Acid site densities were calculated based on eq 2,
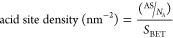
2where AS is the number of acid sites (Brønsted, Lewis, or both) based on catalyst mass, *N_A_* is Avogadro’s number, and *S*_BET_ is catalyst surface area as determined from N_2_ physisorption measurements.

X-ray diffraction (XRD) was employed to evaluate catalyst crystallinity and phases present with a PANlytical Empyrean powder diffractometer using Cu Kα radiation (λ = 0.154 nm) with a step size of 0.013° between 2θ = 10 and 80°. The percentage of tetragonal phase was determined from the ratio of the integrated diffraction intensities corresponding to tetragonal (2θ = 30.2°) and monoclinic (2θ = 28.2° and 31.4°) ZrO_2_, as described elsewhere.^52^

X-ray photoelectron spectroscopy (XPS) was performed using a Thermo Scientific ESCALAB Xi^+^ photoelectron spectrometer furnished with a monochromated Al Kα source (1486.68 eV) and charge compensator. Survey spectra were recorded from 0 to 800 eV at a pass energy of 40 eV with a step size of 0.5 eV. High-resolution XPS spectra of C 1s, O 1s, S 2p, and Zr 3d were obtained using 10–40 eV pass energies and step sizes of 0.05–0.1 eV.

The relative atomic composition (S and H wt %) of catalysts was determined through elemental analysis (EA) using a Carlo Erba 1108 Combustion Analyzer (Atlantic Microlabs, Inc., Norcross, GA, U.S.A.). Prior to analysis, samples were dried at 150 °C under vacuum for 2 h to remove physisorbed water.

### Catalytic Reactions

Sorbitol dehydration reactions were performed in the liquid phase in glass tubes exposed to ambient air, immersed in a preheated oil bath. The SZ catalyst (25 mg) and sorbitol (0.5 mmol, 91 mg) were added to the tubes and allowed to reach reaction temperature under magnetic stirring (450 rpm). After the allotted time, water was added to quench the reaction and the mixture was then briefly sonicated. The catalyst was separated by filtration through a cellulose membrane filter (Whatman ME 25/21 ST) and the filter paper upon which the catalyst was collected was further washed with water. (*Due to the difficulty of sampling under the melt-phase reaction conditions, each data point in time-resolved experiments corresponds to an independent experiment*.) The reaction solution was concentrated under reduced pressure and analyzed via quantitative ^13^C NMR (nuclear magnetic resonance) on a Bruker Avance NEO 500 MHz spectrometer equipped with a 5 mm CryoProbe Prodigy BBO probe. Spectra were recorded using a 60 s recycle delay in 10% D_2_O with ethanol as reference (58.05 ppm CH_2_) and internal standard. Conversions, yields, and selectivity were based on integrations obtained from quantitative ^13^C NMR shown in eqs 3–5,
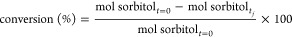
3
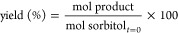
4
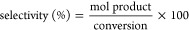
5where *t =* 0 and *t_f_* are the initial and final reaction times, respectively. Because each reaction species has multiple carbon signals, corresponding peak integrations often differed by ∼1–2% and were averaged. In samples with complete conversion, 10 mM GdCl_3_ was used as a paramagnetic relaxation agent to expedite analysis, enabling a 7.5× reduction in analysis time with comparable signal-to-noise. An inversion recovery experiment (Figure S1) was conducted to estimate the longest *T*_1_ present to determine the necessary recycle delay for accurate quantification when using GdCl_3_.^53^

### Total Dissolved Solids (TDS) Analysis

A total dissolved solids experiment was performed to ascertain the relative contribution of species not observable via ^13^C NMR. Prior to reaction, the masses of a cellulose membrane filter (Whatman ME 25/21 ST) and empty 25 mL round-bottom reaction flask were recorded. The catalyst (0.1-SZ-525) and sorbitol (0.5 mmol) were weighed and added to the reaction flask in a preheated oil bath at 150 °C with magnetic stirring. After the allotted time, the reaction mixture was quenched with water and the catalyst was separated using the pre-weighed filter. The filter and catalyst were then dried under reduced pressure to obtain the post-reaction catalyst mass. Similarly, the reaction mixture was concentrated to dryness in the pre-weighed reaction flask under reduced pressure for 48 h, after which the final mass was recorded. Quantification of reaction species was achieved using ^13^C NMR in 10% D_2_O with ethanol as an internal standard as described above. The total mass of the post-reaction material was used in conjunction with quantification data to determine the mass of species not accounted for in NMR.

### Catalyst Recycling and Regeneration

Recycling tests were conducted at 150 °C for 5 min. After the reaction, the sample was cooled and quenched with MeOH and then centrifuged at 6000 rpm for 10 min. The solution was decanted, and the catalyst was washed and centrifuged once more. MeOH was used as solvent in work up and NMR preparation to minimize potential sulfate leaching. The two MeOH fractions were combined, concentrated under reduced pressure, filtered through a 0.2 μm syringe filter, and prepared for ^13^C NMR analysis without GdCl_3_ as described above. The spent catalyst was dried at 120 °C overnight and regenerated at 500 °C for 5 h under flowing air to remove organic deposits. A small amount of catalyst (<5% by weight) was lost during each recovery process, and therefore the amount of sorbitol was scaled down in subsequent trials to maintain the initial catalyst:substrate ratio.

### Isosorbide Isolation

Purification and isolation of isosorbide was conducted using a solid–liquid extraction on dry, post-reaction samples. Chloroform (CHCl_3_, 10 mL) was added to the product after which the mixture was vigorously shaken, sonicated, and decanted. Two identical washing procedures were carried out, and the entire liquid volume was subsequently filtered to remove trace suspended humins and catalyst with an isosorbide recovery of 96% as determined by quantitative ^13^C NMR. Storage of the isosorbide-CHCl_3_ solution at −25 °C resulted in deposition of colorless crystalline needles after 72 h.

## Results and Discussion

### Catalyst Synthesis, Activity, and Reaction Characterization

**Scheme 2:**
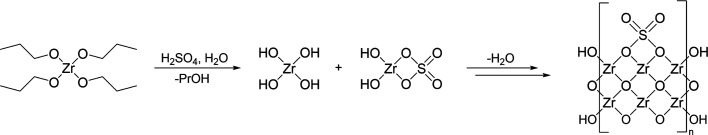
Sol–Gel Synthesis of SZ Displaying Hydrolysis and Polycondensation Steps Balanced stoichiometry is omitted for ease of representation.

The sol–gel synthesis used herein involves the hydrolysis of a Zr alkoxide with aqueous sulfuric acid to yield *in situ* Zr(OH)_4_, which then forms a colloidal network, or ‘sol’, under strong stirring. This is followed by hydroxide polycondensation to form interconnected Zr–O–Zr bonds, generating a three-dimensional porous ‘gel’ network as shown in Scheme 2. After drying and calcining, the desired SZ catalyst is obtained.^54,55^ The sol–gel synthesis is a ‘one-step’ process as the aqueous H_2_SO_4_ hydrolysis catalyst also serves as an in situ sulfating agent, known to produce SZs with the highest relative surface areas, sulfate loadings, and activities.^46,56−58^

Preliminary efforts followed a commonly reported route wherein H_2_SO_4_ is added to the metal alkoxide (‘acid-to-alkoxide’).^59^ However, this order of addition resulted in poor dispersion due to formation of a thick top layer of Zr hydroxide, impeding stirring and hindering the reaction of acid with free alkoxide. Further, due to the moisture sensitivity of Zr *n*-propoxide, hydrolysis was triggered by ambient moisture prior to acid addition, effectively removing control over this crucial first step and therefore morphology. Our modified synthesis (‘alkoxide-to-acid’, Figure S2) reverses the addition order and localizes hydrolysis to the phase interface by increasing the amount of water available to molecular Zr. The impact of the synthesis variables on bulk SZ parameters including surface area and porosity will be discussed below.

**Figure 1:**
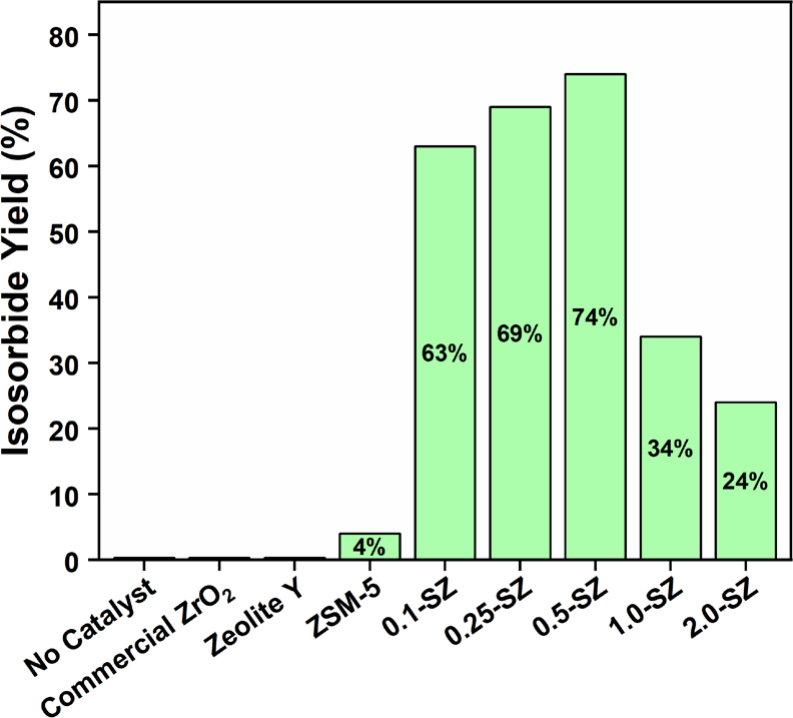
Isosorbide yields obtained with commercial ZrO_2_, zeolites, and 5 different SZ catalysts prepared according to the sol–gel synthesis procedure. Reaction conditions: 150 °C, 2 h, 25 mg catalyst, 91 mg (0.5 mmol) sorbitol. All SZ catalysts were calcined at 625 °C and exhibited complete conversion. Isosorbide yields were calculated from ^13^C NMR data with ethanol as internal standard.

For initial screening, five SZ catalysts were prepared according to the above modification varying S/Zr from 0.1 to 2 (‘alkoxide-to-acid’, calcined at 625 °C) and tested for their sorbitol dehydration activity. For comparison, zeolites (available commercially) and unmodified ZrO_2_ (control) were also evaluated. The reaction was run in the liquid substrate melt (sorbitol m.p. 95 °C) without added solvent. Furthermore, dehydrations were conducted under an ambient atmosphere (open to air) without pressure or vacuum setups. Upon scale-up, operating in this manner should be safer and limit total energy expenditure. As shown in Figure 1 and Table S1, conversion was poor over zeolite Y (7%) and ZSM-5 (16%), activity differences consistent with previous reports concerning Si:Al.^24,60^ SZ catalysts dramatically outperformed the zeolites under the reaction conditions, with 0.5-SZ found to be most selective giving 74% yield of isosorbide. All SZ-625 catalysts achieved complete conversion of sorbitol in the allotted 2 h reaction time, and isosorbide yield improved from 63% for 0.1-SZ to 74% over 0.5-SZ. Yields dropped precipitously when S/Zr ≥ 1.0, likely a result of substantial oligomerization as evidenced by the dark brown color of the post-reaction solutions.^61,62^ The unmodified ZrO_2_ control was inactive.

**Figure 2:**
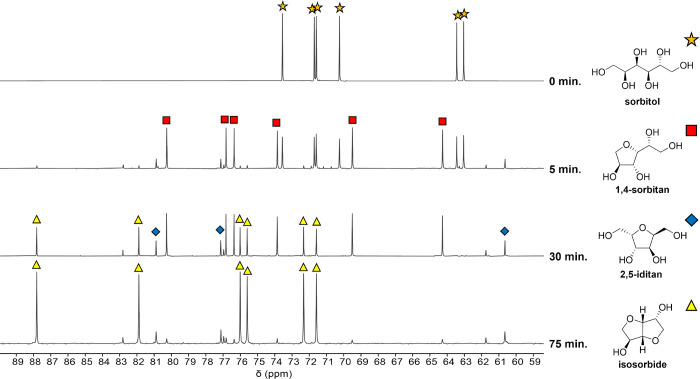
Stacked ^13^C NMR spectra of sorbitol dehydration product mixtures at selected reaction times. Resonances characteristic of major species are indicated. Reaction conditions: 150 °C, 25 mg 0.5-SZ-625 catalyst, 91 mg (0.5 mmol) sorbitol.

For product analysis, ^13^C NMR spectroscopy was employed in place of high-performance liquid chromatography (HPLC) since several anhydrohexitol intermediates (Scheme 1) could not be effectively resolved via HPLC (Figure S3), consistent with previous reports.^60,61,63^ The ^13^C NMR spectra were used to monitor sorbitol, anhydrohexitols, and isosorbide, allowing complete resolution and quantification of all species as shown in Figure 2. Full assignment of all minor species across additional reaction times is available in the Supporting Information (Figure S4). Four anhydrohexitol intermediates were detected, the 5-membered cyclic ethers: 1,4-sorbitan, 3,6-sorbitan, 2,5-mannitan, and 2,5-iditan. Selective production of 1,4- and 3,6-sorbitan (collectively referred to as 1,4-AHSO) intermediates is key to high product yields since only these two anhydrohexitols undergo subsequent dehydration to isosorbide. In contrast, 2,5-mannitan and 2,5-iditan (collectively termed 2,5-AHSO) are dead-end byproducts (Scheme 1). Equally important to constraining 2,5-AHSO production is limiting the formation of humins, darkly colored oligomeric products that were not observable in the ^13^C NMR spectra in 10% D_2_O. As a result, in many experiments, some mass remained unaccounted for after NMR quantification of C6 products. Mass balance closure was achieved upon attribution to humins following a total dissolved solids (TDS) analysis (Table S2).

**Figure 3:**
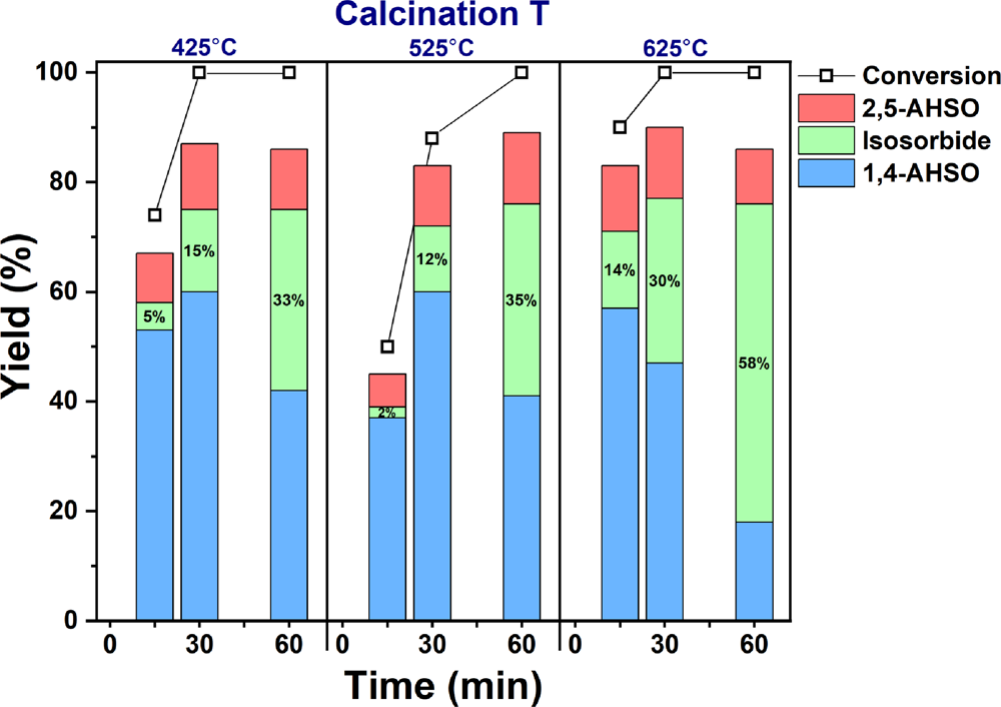
Sorbitol dehydration activities of 0.5-SZ catalysts calcined at 425, 525, and 625 °C. The unaccounted-for mass was attributed to humins formation as confirmed by total dissolved solids analysis (Table S2). 1,4-AHSO = 1,4-sorbitan and 3,6-sorbitan; 2,5-AHSO = 2,5-mannitan and 2,5-iditan. Reaction conditions: 150 °C, 25 mg 0.5-SZ catalyst (calcined at 425, 525, or 625 °C), 91 mg (0.5 mmol) sorbitol. Conversion and yields determined from the ^13^C NMR spectra with ethanol as internal standard.

Beyond the effect of just S/Zr, the calcination temperature can impact catalyst acidity and crystallinity. Previous SZ studies have shown that low temperature calcination may not sufficiently induce active site formation, whereas very high temperature can cause deleterious loss of sulfates and activity.^58,64^ With this in mind, we employed a range of calcination temperatures, 425, 525, and 625 °C, on the optimum catalyst from screening (0.5-SZ, Figure 1) to investigate its effect in isosorbide formation. Regardless of catalyst calcination temperature, all reactions reached complete conversion of sorbitol within 1 h at 150 °C (Figure 3), featuring 1,4-AHSO as the major intermediate. Nevertheless, in terms of isosorbide formation, 0.5-SZ-625 gave the most rapid rate of production at 11.6 mmol h^–1^ g^–1^ (mol isosorbide / h / g catalyst), whereas 0.5-SZ-425 and 0.5-SZ-525 were slower at 6.6 and 7.0 mmol h^–1^ g^–1^, respectively.

**Figure 4:**
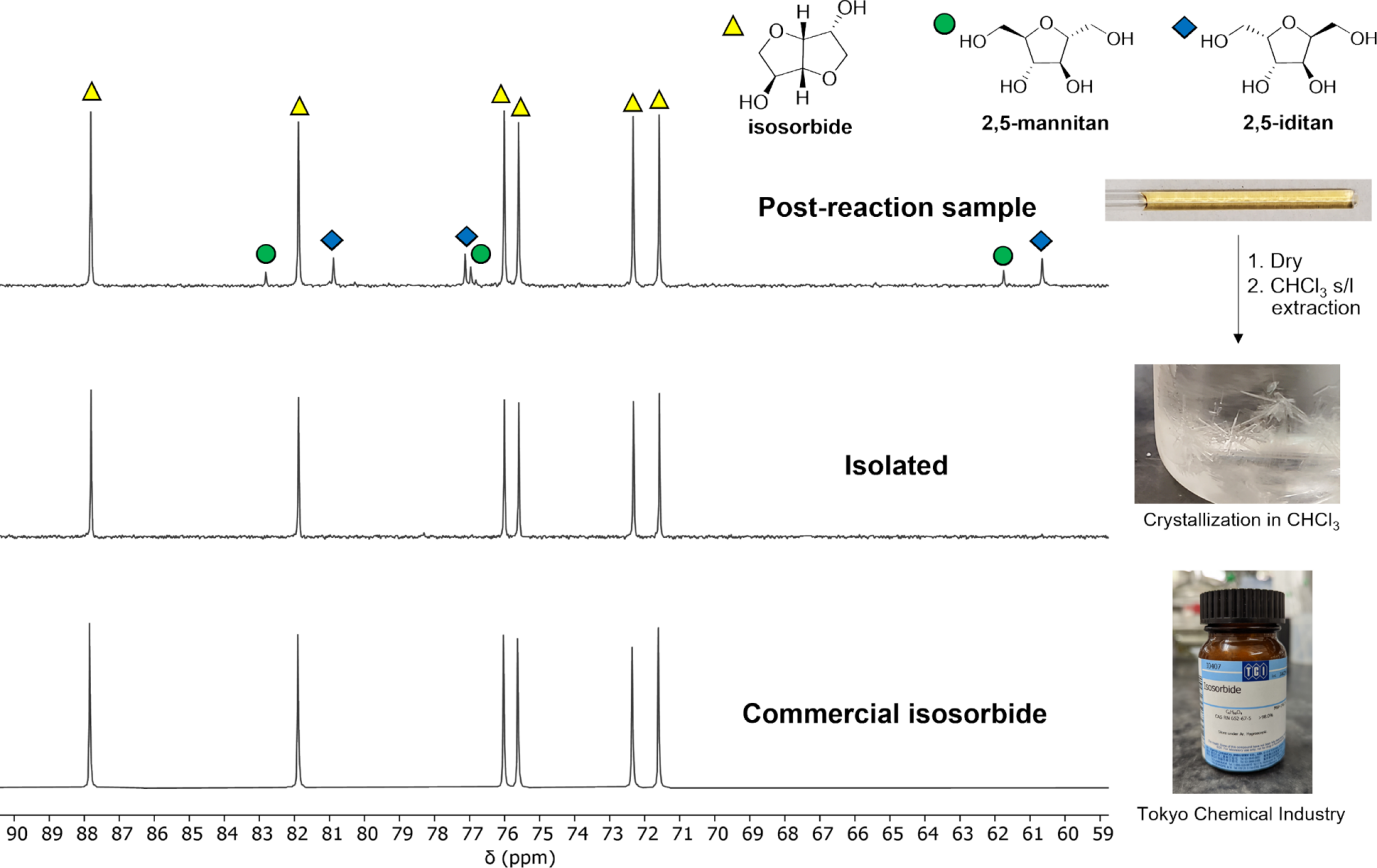
Top: ^13^C NMR spectrum (10% D_2_O) of the product mixture after a reaction carried out under the following conditions: 160 °C, 2 h, 25 mg 0.5-SZ-625 catalyst, 91 mg (0.5 mmol) sorbitol. Middle: ^13^C NMR spectrum of the isosorbide extracted from the above mixture with CHCl_3_ (3 × 10 mL). Bottom: ^13^C NMR spectrum of a commercial sample of isosorbide.

While a heterogeneous catalyst such as SZ enables facile separation of the catalyst from the reaction mixture, the isolation and purification of isosorbide from the reaction byproducts is also important.^43^ Separations account for over 40% of the chemical industry’s total energy consumption; therefore, less energy-demanding alternatives to traditional thermal methods such as distillation are desirable.^65^ After complete conversion of all 1,4-AHSO intermediate over 0.5-SZ-625 (160 °C), the product mixture contained isosorbide (70%), 2,5-AHSO isomers (6%), and humins (24%, via TDS) as shown in Figure 4. Separation of isosorbide from a reaction mixture containing byproducts with similar chemical properties is challenging.^66^ Nevertheless, extraction of isosorbide from the solid product mixture proved quite successful. Common polar organic solvents ethyl acetate and acetone were not effective, dissolving 2,5-AHSO byproducts as well; however, chloroform was found to be selective for isosorbide, achieving 96% recovery after three extractions (10 mL each). Isolated isosorbide (67% yield) was obtained in good purity according to its ^13^C NMR spectrum, matching that of a commercial sample (Figure 4).

### Kinetic Analysis and Modeling

Quantitative ^13^C NMR provides a powerful technique in this instance to study the chemical kinetics of sorbitol dehydration to isosorbide over SZ because, in addition to reactants and products, the evolution and disappearance of intermediates can be discerned. A simplified kinetic mechanism is shown in Table 1, consisting of five irreversible reactions between five species: sorbitol, 1-4-AHSO, 2,5-AHSO, isosorbide, and humins. The model therefore has five reactions with five rate constants, and the rate constants are assumed to have Arrhenius dependence on temperature (eq 6)
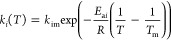
6where *k*_im_ is the rate constant’s pre-exponential factor at the chosen mean temperature, *T*_m_ = 150 °C, and *E*_a*i*_ is the activation energy for reaction *i*. Measurements of the temporal species concentrations from isothermal batch experiments were conducted at four different temperatures: 140, 150, 160, and 170 °C (Table S3 and Figure S5). The pre-exponential factors and activation energies were estimated by minimizing the sum of squares of the five species’ measurement errors for all four temperature experiments simultaneously. Approximate 95% confidence intervals for the estimated parameters, and the sizes of these confidence intervals indicate whether the set of experimental runs contain sufficient information to estimate the model parameters.

**Table 1:**
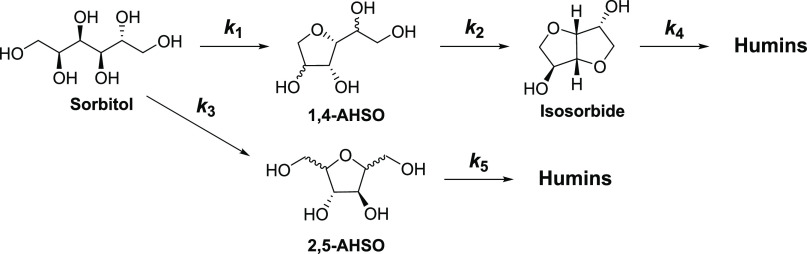
Rate Constants (s^–1^) and Activation Energies for the Temporal Data Obtained for Reaction of Sorbitol over 0.5-SZ-625 at Various Temperatures

*T*	*k*_1_	*k*_2_	*k*_3_	*k*_4_	*k*_5_	*k*_1_/*k*_3_
140 °C	4.3	0.82	1.0	0.040	0.27	4.2
150 °C	8.5 ± 2.9	1.5 ± 0.3	1.8 ± 1.2	0.070 ± 0.061	0.48 ± 1.0	4.7
160 °C	16	2.7	3.1	0.12	0.82	5.2
170 °C	30	4.7	5.3	0.20	1.4	5.8
*E*_a_ (kJ/mol)	99 ± 42	88 ± 32				

**Figure 5:**
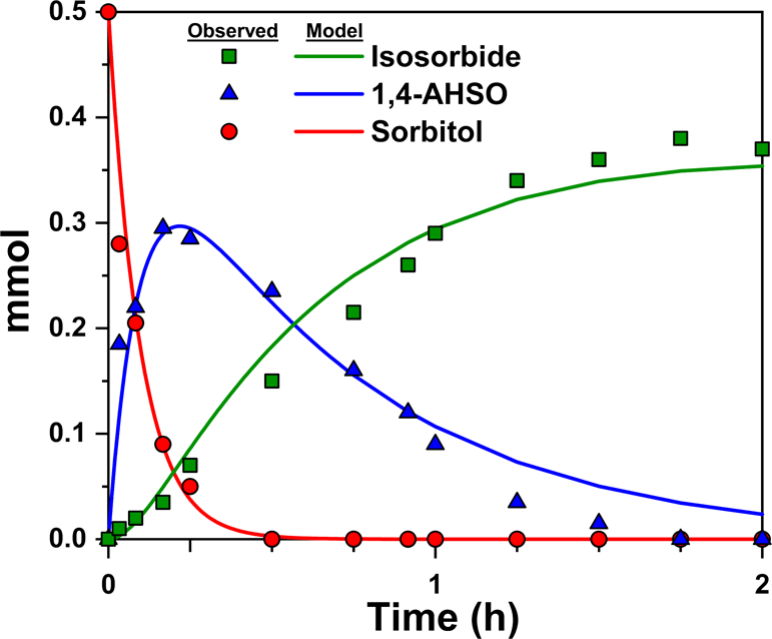
Shown are the fits of the kinetics model using the rate constants shown in Table 1 for 150 °C to the experimental data points. Reaction conditions: 150 °C, 25 mg 0.5-SZ-625 catalyst, 0.5 mmol (91 mg) sorbitol. The data indicating the three species shown were obtained from an independent experiment for each reaction time.

The first three species concentrations from a typical experiment are shown in Figure 5. From the scheme in Table 1, there are five pre-exponential factors and activation energies to estimate. The resulting large confidence intervals in the five estimated activation energies clearly indicated that the experimental measurements did not contain enough information to estimate all five activation energies. The problem was therefore reduced by estimating the activation energies of only the first two reactions (*E*_a1–2_) with other three activation energies (*E*_a3–5_) fixed at nominal values to give the estimated parameters shown in Table 1. The five pre-exponential factors (at *T_m_* = 150 °C) and two activation energies then have reasonably tight confidence intervals as shown in Table 1. The fit to the measurements for all five species at all four temperature runs was similar to that shown in Figure 5. We conclude that this set of experiments can be represented by the chosen simplified mechanism, and the seven kinetic parameters (five rate constants and two activation energies) can be estimated with reasonably tight confidence intervals from the available species measurements. Consistent with previous investigations of sorbitol dehydration kinetics, the rate-limiting step (*k*_2_) involves the annulation of 1,4-AHSO via cyclodehydration to generate isosorbide.^61,62^ This results in a buildup of the 1,4-AHSO intermediates, yielding curves of temporal reaction data typical of the form shown in Figure 5. Notably, the *k*_1_/*k*_3_ ratio provides an important measure of desired pathway selectivity and increases with temperature (Table 1). However, formation of humins from isosorbide (*k*_4_) and 2,5-AHSO (*k*_5_) also increased; therefore, reaction temperature must be carefully considered to balance product formation in the face of counterproductive oligomerization.

### Catalyst Characterization

#### Surface Area, Crystallinity, and Morphology

Altering the hydrolysis rate in sol–gel syntheses enables control over particle morphology wherein a rapid hydrolysis (first step, Scheme 2) generates high surface area materials. Besides water, an acidic environment can beneficially impact this rate and resulting surface area;^58,67^ however, the conditions for obtaining the highest surface area materials and how the surface areas are impacted by S/Zr can vary widely depending on the synthetic methodology. A range of S/Zr ratios and three different calcination temperatures were investigated, and the characterizations described below consider both S/Zr and calcination conditions.

**Figure 6:**
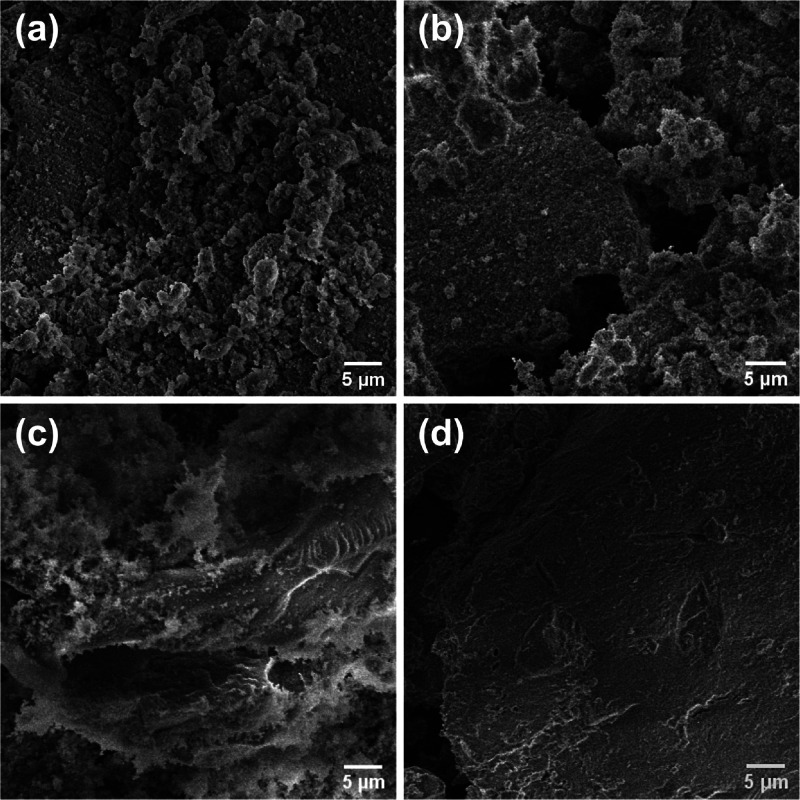
SEM images of (a) 0.1-SZ, (b) 0.5-SZ, (c) 1.0-SZ, and (d) 2.0-SZ catalysts calcined at 625 °C.

Surface areas (*S*_BET_) and pore volumes (*V*_p_) of the resulting materials were determined by N_2_ physisorption. A non-sulfated sample (NS) was used as a control for comparisons to the SZs. A linear relationship was obtained in the BET transform plot (Figure S6), where *S*_BET_ in NS was determined to be 74 m^2^/g as shown in Table 2. Upon initial acid introduction (0.1-SZ), a marked increase in *S*_BET_ (133 m^2^/g) was achieved, consistent with the positive effect in catalyzing hydrolysis.^58,67^ However, each subsequent increase in H_2_SO_4_ concentration produced a corresponding decrease to *S*_BET_ (Table 2), a trend also readily visualized in SEM imaging. As S/Zr increased, the resulting SZs formed progressively larger particles with smoother surfaces, reducing *S*_BET_ (Figure 6). Increased calcination temperature also led to decreased surface areas via catalyst sintering (Table S4), where materials with high *S*_BET_ (low S/Zr) were most susceptible. Despite this trend, low and moderate acid quantities of S/Zr ≤ 0.5 produced SZs with surface areas superior to the sample synthesized with water alone (NS). This suggests that optimal hydrolysis occurs at low acid concentration with Zr in excess but is negatively impacted at higher concentrations.

**Table 2:** Structural and Physical Properties of SZ Catalysts Calcined at 625 °C with Varying *S*/*Z* Ratios

catalyst	*S*_BET_^a^ (m^2^/g)	pore volume^b^*V*_p_ (mL/g)	pore diameter^c^*D*_p_ (nm)	tetragonal phase^d^ (%)
NS	74	0.60	31.2	52
0.1-SZ	133	0.85	23.8	83
0.25-SZ	127	0.69	21.3	81
0.5-SZ	81	0.37	18.6	69
1.0-SZ	44	0.16	18.3	Amorphous
2.0-SZ	29	0.08	7.5	Amorphous

a Surface area (*S*_BET_) determined from BET analysis on adsorption branch.

b Calculated from BJH analysis on the desorption branch.

c Average mesopore diameter.

d The percentage of the tetragonal phase was determined from the ratio of the integrated intensities of diffraction angles corresponding to tetragonal (2θ = 30.2°) and monoclinic (2θ = 28.2° and 31.4°) zirconia, as described elsewhere.^52^

**Figure 7:**
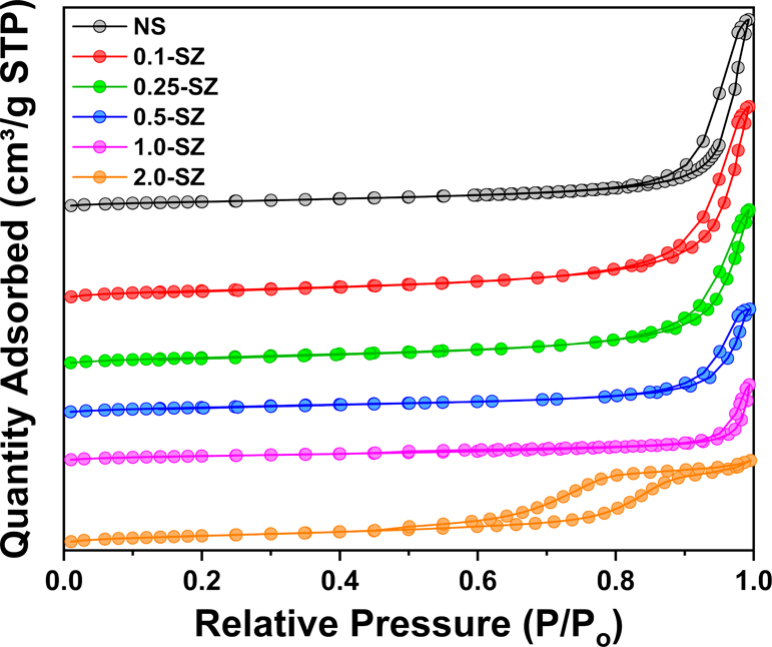
N_2_ adsorption–desorption isotherms of SZ catalysts following 625 °C activation.

Adsorption–desorption behavior shown in Figure 7 indicates that all samples were mesoporous in nature, exhibiting isotherms of type IV (IUPAC). Increasingly corrosive conditions restricted large pore formation in SZs, where pore volumes and diameters decreased with S/Zr (Table 2). Although often favorable to *S*_BET_, even low acid concentrations markedly affected pore development in SZs and gave smaller average pore diameters than NS. From corresponding pore size distributions, most pores present in NS were in the upper mesopore range (20–50 nm), whereas in 0.5-SZ for example, the majority were between 2 and 5 nm (Figure S7a). Despite the two having similar *S*_BET_ measurements, NS possessed a pore volume (0.60 mL/g) nearly twice that of 0.5-SZ (0.37 mL/g).

Thermal conditioning via calcination facilitates long-range ordering in SZs as the amorphous materials transition to the tetragonal (T) and monoclinic (M) ZrO_2_ phases; however, surface sulfates are known to restrict the T → M phase transition.^68^ Therefore, SZs typically contain a greater proportion of the metastable T phase compared to unmodified ZrO_2_. Accordingly, powder X-ray diffractograms (Figure S8a) show NS to have a greater contribution from the M phase than crystalline SZs, while 1.0-and 2.0-SZ remained amorphous even after 625 °C calcination. Table 2 shows the different phase distributions in SZs; the NS sample has a roughly equal proportion of T and M while sulfated samples 0.1-, 0.25-, and 0.5-SZ are all at least two-thirds T. Surprisingly, the lowest sulfate loading of 0.1-SZ was most effective at restricting the T → M transition (0.1-SZ-525, 100% T, Table S4), while increasing sulfation in 0.25- and 0.5-SZ resulted in progressively less T and more M phases.

#### Surface Sulfates and Acidity

**Figure 8:**
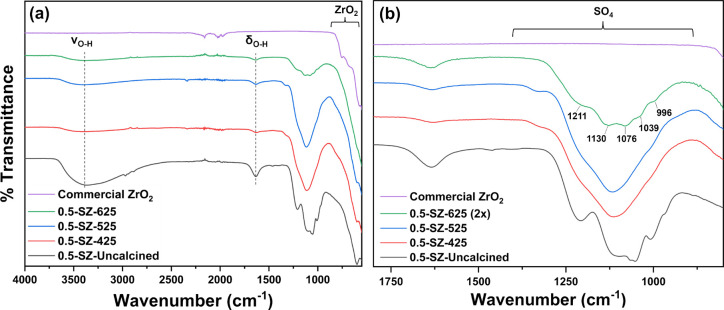
FTIR spectra of ZrO_2_ and 0.5-SZ catalysts monitoring the effect of calcination across the (a) full spectrum range and (b) sulfate region.

Catalyst surfaces were investigated using FTIR to probe relevant surface moieties including sulfate structure and coordination mode as well as how these are affected by calcination temperature. Figure 8a displays a wide high-energy absorption centered around 3400 cm^–1^ attributed to ν_O-H_ stretching modes from surface hydroxyl groups and adsorbed water, while a band at 1630 cm^–1^ was ascribed to δ_O-H_ bending mode of water. Calcination led to a decrease in these intensities via dehydration and olation. These absorbances were not detected in ZrO_2_, however, consistent with previous descriptions of post-calcination rehydration of SZs and indicating that sulfate groups induce this hygroscopicity. Rehydration results in S=O bonds with increased ionic character and decreases their corresponding bond energies.^69,70^ Strong, broad absorptions present in the region of 1360–880 cm^–1^ prior to calcination indicate successful decoration of sulfate groups onto the surface. Thermal treatment up to 525 °C did not result in significant sulfate decomposition as the respective IR intensities remained largely unchanged. However, resolution was poor due to high sulfate coverage as suggested by S elemental analysis (Table S5). Additionally, the amorphous ZrO_2_ surfaces from XRD analysis allow a multitude of possible coordination environments, which can induce small bond energy perturbations, broadening absorptions and restricting detailed assignments (Table S4 and Figure S8b). An abrupt decrease in sulfate intensity was observed upon calcination at 625 °C (Figure 8a), implying that significant decomposition of sulfate groups initiates between 525 and 625 °C in 0.5-SZ.

A variety of surface sulfate geometries in SZ have been reported, including *C*_2v_ chelating or bridging bidentate,^71^*C*_3v_ tridentate,^72^ and pyrosulfate structures.^42^ Pyrosulfate species have been previously attributed to strong acidity in SZ but the characteristic IR frequency (>1400 cm^–1^)^73^ was not observed in any sample. Since pyrosulfates are readily hydrolyzed under the analysis and reaction conditions, their presence here is unlikely.^74,75^ Residual sulfuric acid with its potential capacity for homogeneous dehydration is also doubtful since the calcination temperatures are significantly above its boiling point (337 °C). Furthermore, the distinctive asymmetric ν_S=O_ band of free H_2_SO_4_ (1360–1370 cm^–1^) was absent in all FTIR spectra.^76−78^ In addition, the kinetics and intermediates observed in the present case, specifically in *k*_1_/*k*_2_ and 3,6-sorbitan, show significant differences from those seen when employing H_2_SO_4_ as a catalyst.^63^ Absorptions located at 1210, 1130, 1076–1039, and 996 cm^–1^ correspond to asymmetric and symmetric ν_S=O_ and ν_S–O_ stretching modes, respectively, consistent with a chelating bidentate sulfate structure (0.5-SZ-625, Figure 8b).^79,80^ The presence of multiple asymmetric ν_S–O_ (1076 and 1039 cm^–1^) has been observed previously and attributed to one initial absorbance mode splitting into two following activation.^69^ Interestingly, a separate infrared analysis of pure T phase SZ recorded a distinct band at 1075 cm^–1^, yet absent in the M spectrum.^81^ On the basis of these observations and together with the spectra in Figure 8b, we propose the presence of five total sulfate infrared absorptions to be the result of sulfate coordination to a biphasic (T and M ZrO_2_) material, such as in 0.5-SZ-625. The two crystalline phases and their relative proportion could potentially impact localized acidity and surface activity.

To further explore the surface acidity, the total loading and strength of acid sites in SZ catalysts were investigated by NH_3_-TPD. Figure S9a shows NH_3_ desorption patterns for SZs and illustrates that all samples primarily contain weak acid sites, reflected by their low temperature maximums. Sulfation did not significantly affect acid site strength given the similarities in NH_3_ desorption behavior between NS and (0.1–0.5)-SZ; however, some stronger acid sites were generated in 1.0-SZ. Listed in Table 3, nearly all SZ catalysts contained higher acid loadings than their NS counterpart, where increasing S/Zr generally produced a corresponding rise in acid site formation except 1.0-SZ, which will be discussed in more detail later. Due to handling difficulty and poor product selectivity, 2.0-SZ catalysts were not investigated further.

**Table 3:** Acid Properties of Selected Catalysts

catalyst	acid site loading^a^ (μmol NH_3_/g)	Brønsted/Lewis ratio^b^	total acid site density^c^ (nm^–2^)
NS-625	228	0.01	1.83
0.1-SZ-625	371	1.04	1.68
0.25-SZ-625	420	0.73	1.99
0.5-SZ-425	470	0.31	3.21
0.5-SZ-525	428	0.93	3.10
0.5-SZ-625	510	1.16	3.79
1.0-SZ-625	132	0.41	1.81

a Temperature-programmed desorption of NH_3_.

b FTIR spectra of adsorbed pyridine.

c Determined from NH_3_-TPD and *S*_BET_, see eq 2.

The type and proportion of acid sites, whether Brønsted or Lewis, can dictate catalyst activity. This property was investigated via FTIR spectra of adsorbed pyridine (Table 3, Figure S10). Sulfation is apparently required for generation of Brønsted acidity, evidenced by the near-zero Brønsted/Lewis (B/L) value of NS. Crucially, varying the temperature of thermal treatment imparted a great degree of control over the acid site identities of the 0.5-SZs (Figure S10). Low temperature calcination resulted in mostly Lewis acid sites (425 °C, B/L = 0.31), but as the calcination temperature increased, so too did the proportion of Brønsted acid sites, eventually resulting in a majority of Brønsted sites following 625 °C activation (B/L > 1, Table 3). Remarkably, tunability of the B/L character with temperature in 0.5-SZs was achieved while total acid loading remained relatively constant (Table 3 and Figure S9b). To better understand the emerging relationship between calcination temperature and acid character, TGA analysis was conducted on an uncalcined pre-catalyst sample to simulate thermal activation in synthesis (Figure S11). Two distinct weight loss features were observed. The first, centered around 120 °C, is primarily due to evolution of physisorbed water with some *n*-propanol also possible. A high-temperature feature centered around 675 °C is ascribed to sulfate decomposition.^82^ Since sulfate loss begins around 575 °C, most sulfates are likely preserved in lower temperature activation (425 or 525 °C). This is consistent with earlier FTIR spectra (Figure 8) and elemental analyses (Table S5) wherein sulfate region intensity and S wt % decreased significantly only upon activation at 625 °C. Interestingly, increased sulfate loss from these catalysts was accompanied by a concomitant rise in the number of Brønsted acid sites. This phenomenon can be rationalized through two mechanisms, potentially working in tandem. First, sulfate loss opens up a coordination site to Lewis acidic Zr^4+^, allowing rehydration to generate a Brønsted site.^64^ Second, this decreased sulfate presence reduces surface strain and enables crystallization to proceed more readily in 0.5-SZ-625, generating bridging hydroxyls through olation that have higher surface density in M-ZrO_2_;^83^ a process restricted in amorphous 0.5-SZs calcined at lower temperatures (Table S4). In summary, increasing calcination temperatures can reduce superfluous sulfate coverage and generate reactive surface hydroxyl groups, resulting in higher B/L.

**Figure 9:**
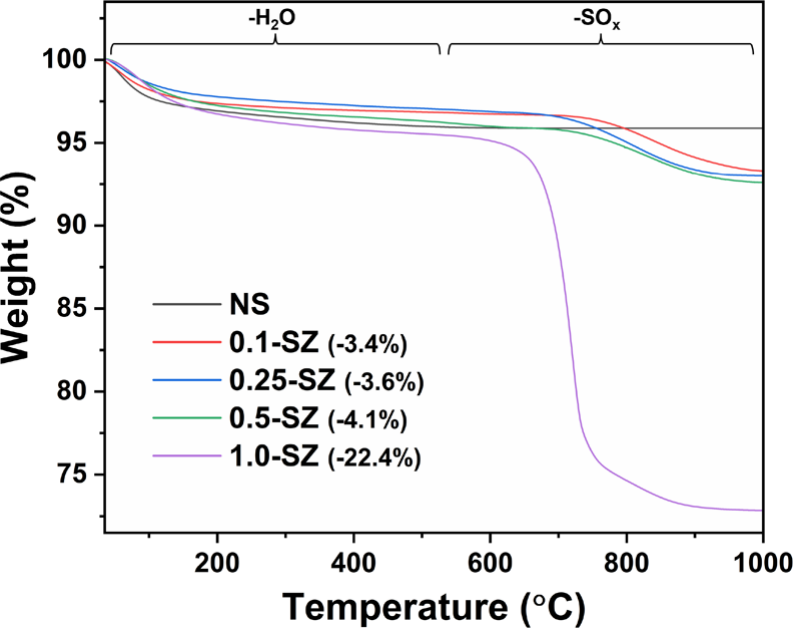
TGA analysis of SZ catalysts calcined at 625 °C with varying S/Zr. Percentages in parentheses correspond to weight loss in the sulfate region.

TGA following high-temperature calcination (Figure 9) also confirmed excellent thermal stabilities of the sol–gel SZs. Like the uncalcined sample (Figure S11), two features are presented. A low-temperature feature is assigned to water loss. Not present in the NS trace (Figure 9), the subsequent high-temperature feature (600–900 °C) in SZs is again attributed to sulfate decomposition, typically as SO_2_.^84−86^ The calculated mass losses in the sulfate region were in good agreement with S composition from elemental analysis (Table S5; SO_2_, ∼50 wt % S). However, the behavior of sulfate decomposition in 1.0-SZ-625 initiated at a lower temperature (centered around 718 °C), indicating that Zr(SO_4_)_2_ instead was produced in synthesis, as described by Hino et al.^70^ Upon surface saturation, the thermal mechanisms that bolster acidity are severely impacted at such high S loading (∼13 wt %), preventing crystallization and blocking potential acid sites. These two factors combine to produce a relatively poor acid loading (Table 3). Formation of Zr(SO_4_)_2_ in synthesis of 1.0-SZ could allow some excess H_2_SO_4_ (b.p. 337 °C) to remain on the surface after low temperature (425 and 525 °C) calcination, a feature corroborated by higher Brønsted loadings in comparison to 1.0-SZ-625 (Table S6).

### Structure–Function Relationship

At the molecular level, sorbitol dehydration over SZ requires Brønsted acid sites, as purely Lewis acidic, non-sulfated (NS) ZrO_2_ yielded no cyclodehydration species. Through sulfation, Brønsted acid centers were generated in all SZs (Table S6), thereby enabling protonation and resulting in successful catalysis of isosorbide formation (Table S1). As the S/Zr ratio increased in 0.25- and 0.5-SZ, acid loadings increased while *S*_BET_ simultaneously decreased, leading to increased acid site densities (Table 3) and a steadily improved product yield (74%, Figure 1). This positive trend in performance reverses upon higher sulfation resulting in low isosorbide yields (24–34%) over (1.0-2.0)-SZ from increased humin formation. Furthermore, despite the substantially different acid properties of 1.0-SZ-525 and 1.0-SZ-625 (the former containing over 4-fold higher acid loading and ∼7× Brønsted sites, Table S6), the two catalysts gave remarkably similar product selectivities (Table S1). While sorbitol dehydration under these conditions clearly requires Brønsted acid catalysis, neither Brønsted loading nor total acid density correlate directly with isosorbide yields. Evidently, the underlying relationship(s) is more complex and may depend on more than one catalyst quality.

**Figure 10:**
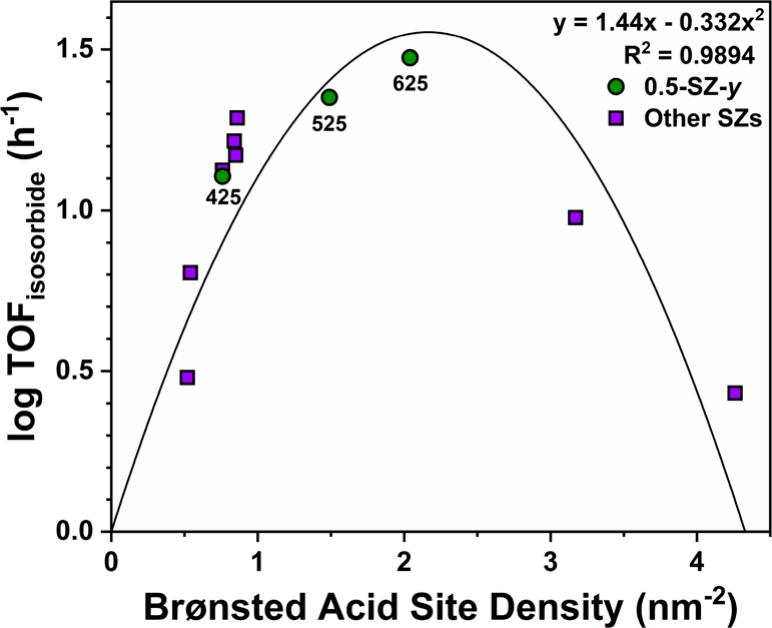
Logarithm-normalized isosorbide turnover frequencies (TOF) and Brønsted acid densities of SZ catalysts. TOF defined in terms of total acidity obtained from NH_3_-TPD. Reaction conditions: 150 °C, 0.5 h, 25 mg catalyst (S/Zr = 0.1–1, calcined 425–625 °C), 0.5 mmol (91 mg) sorbitol. Second-order polynomial of the form *y* = *Ax*^2^ + *Bx* + *C* used for data fitting; *A* = 1.44, *B* = −0.322, *C* = 0, *n* = 11. 0.1-SZ-425 omitted due to low activity.

Upon more closely examining acid-based descriptors and the wide range of surface areas of SZs obtained through the sol–gel synthesis, we found that Brønsted acid surface density (as defined by eq 2) correlates with catalytic activity. This underlying relationship is exhibited in Figure 10. Moderate adjustments to the surface concentration of Brønsted acid sites resulted in remarkable differences in TOF_isosorbide_, exceeding one order of magnitude in some cases. The underlying mathematical relationship that describes the observed data was a second-order polynomial (quadratic) function; TOF_isosorbide_ increases at low Brønsted densities (0.5–1.5 nm^–2^), reaches a maximum at ∼2.25 nm^–2^, and then subsequently inverts and decreases at higher densities. This relationship helps explain the high activity over 0.5-SZ-625 as the density of Brønsted sites on its surface (2.04 nm^–2^) is closest to the calculated maximum.

While the SZ catalytic activity described herein is clearly a function of Brønsted surface density, the cooperative role of Lewis acids in sorbitol dehydration also must be considered.^21,87^ Guo et al. recently observed that the addition of a Lewis acid co-catalyst resulted in higher yields of isosorbide than with pure Brønsted acids. This observation was rationalized through the ability of Lewis sites to interact with reactive hydroxyl groups to prevent undesired reactions and, in doing so, direct Brønsted sites to the appropriate target.^32^ We argue this mechanism of quasi- protection by Lewis acids may also limit decomposition to humins, decreasing oligomerization losses and preserving isosorbide yield. Because sorbitol dehydration primarily proceeds via successive S_N_2 steps at terminal carbons (C1 followed by C6, or vice versa) to generate 1,4-AHSO, both steps rely upon preferential protonation of primary -OH over secondary -OH despite a lower basicity in the former (see proposed mechanism, Scheme S1); Lewis centers can therefore help drive the targeted transformations forward by suppressing others. In a heterogeneous catalyst like SZ, each Lewis site theoretically would be most effective when proximal to a Bronsted acid site, forming a localized Brønsted–Lewis acid pair, or that is, roughly when B/L ≈ 1. Therefore, we argue that in addition to Brønsted surface concentration, the relative proportion of these sites to Lewis sites are two central parameters governing TOF_isosorbide_, a relationship best visualized through varying calcination temperatures for 0.5-SZ catalysts (green circles, Figure 10).

Following the first 100 °C increase in calcination temperature in 0.5-SZ (425 to 525 °C), Brønsted acid site density increased by roughly a factor of two (Table S6). This, along with increased B/L, appreciably impacts activity with TOF_isosorbide_ approximately doubling from 13 to 23 h^–1^. Upon 625 °C calcination, Brønsted density and B/L further increased in part due to the relatively high proportion of monoclinic ZrO_2_ present (31% M, most among 625 °C SZs, Table S3), known to contain a greater concentration of surface -OH than T-ZrO_2_.^83^ The presence of multiple crystalline phases in SZs can impact catalytic activity due to coexistence of different sulfate interactions present on these complex supports and local surface environments, altering acid character and substrate interactions. Consequently, these temperature-induced structural changes brought about further increases in TOF_isosorbide_, reaching its highest value of 30 h^–1^ (0.5-SZ-625, Table S6 and Figure 10).

In summary, each SZ acid site is most effective for sorbitol dehydration when there are (i) an approximately equal proportion of Brønsted and Lewis acid sites (B/L ≈ 1) and (ii) a surface concentration of ∼2 Brønsted centers/nm^2^. The former (i) directs protonation to the desired hydroxyl groups via selective Lewis-based coordination and the latter (ii) optimizes catalyst–substrate interactions to balance adsorption, dehydration, and desorption, while both provide additional function in limiting oligomerization. Brønsted acid density is therefore the main factor to determine surface interactions and propensity for dehydration. When too low, interaction strength and local proton availability are poor, giving reduced activity. On the other hand, when the density is too high, adsorption is too strong and species become vulnerable to oligomerization, which further serves to reduce available active sites. Therefore, the Brønsted density and near-equal proportion of B/L in 0.5-SZ-625 achieve the desired balance between reaction catalysis while limiting losses to humins, attaining the highest TOF_isosorbide_ (Figure 10). This renders the obtained SZs pseudo-bifunctional in nature; synergism between two reactive centers on a single catalyst surface removes the need for a Lewis acid additive through tunability of the B/L acid site ratio. In fact, this simultaneous presence of both Brønsted and Lewis acid sites in SZs, despite neither being exceptionally strong, has been described as the underlying reason behind their so-called ‘superacidic’ nature.^88^

### Catalyst Recycling and Regeneration

A desirable quality of heterogeneous catalysis is the ease of catalyst separation and potential for their reuse, lowering cost and reducing waste.^89^ Several accounts of recyclable SZ catalysts have been published,^59,90−92^ but deactivation, in some instances irreversible, has also been observed. Particularly prevalent in aqueous phase transformations, SZ deactivation can occur via sulfate leaching.^42,93,94^ However in a similar melt-phase system as this, leaching was determined to not be a cause of deactivation.^41^ To ensure the reaction is indeed catalyzed by SZ and eliminate the possibility of a homogeneous contribution via leaching, a set of experiments were conducted using small quantities of H_2_SO_4_ based on S elemental analysis with and without SZ (Figure S12). In both cases over H_2_SO_4_ alone (Figure S12a,b), no conversion was detected. At the same time, the SZ control reaction (Figure S12c) achieved remarkably comparable conversion and selectivity when both catalysts were present (Figure S12d), allowing us to conclude that the reaction is catalyzed principally by SZ.

**Figure 11:**
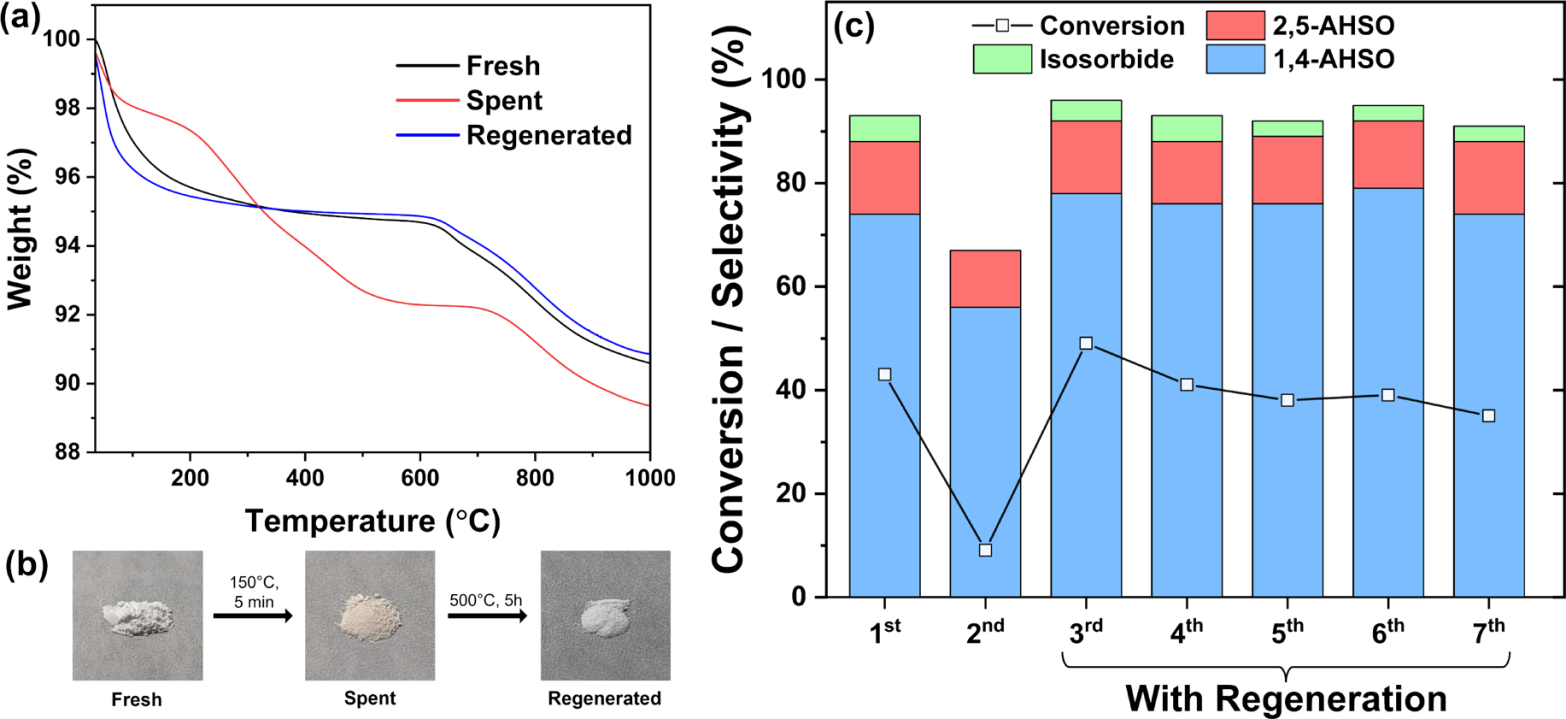
(a) TGA analysis and (b) images of fresh, spent, and regenerated catalysts. Following the reaction, the spent catalyst was obtained after rinsing with MeOH and drying at 120 °C. (c) Catalyst stability with and without regeneration. First trial reaction conditions: 150 °C, 5 min, 50 mg 0.5-SZ-625 catalyst, 1 mmol (182 mg) sorbitol. A small loss in catalyst mass occurred with each use after separation. Subsequent trials were scaled down to maintain the initial catalyst to substrate ratio. Regeneration conditions: 500 °C, 5 h under flowing air. Conversion and selectivities calculated by ^13^C NMR.

Recycling tests began with fresh catalyst under conditions (150 °C, 5 min) to ensure only partial conversion.^95^ After reaction and separation, the spent SZ was used again without additional treatment. Conversion of sorbitol to intermediates and products dropped from 43% with fresh catalyst to just 9% in the following run over the recycled catalyst, indicative of deactivation. The reaction also showed poorer selectivity to desired anhydrohexitol intermediates (1,4-AHSO) and a concomitant increase in humin production with the recycled catalyst (Table S7). TGA analyses of fresh and spent samples (Figure 11a) showed that while the spent catalyst exhibited the typical dehydration and sulfate decomposition regions of the fresh catalyst, an additional weight loss feature was seen between 200 and 500 °C, suggesting thermal elimination of surface organic deposits. In addition, the spent catalyst had also undergone a color change to light brown, suggesting that humins could be a primary source of coke (Figure 11b). From the recorded TGA behavior, oxidative regeneration at 500 °C was deemed sufficient to remove carbonaceous species, which also served to restore the original catalyst color. Indeed, subsequent TGA analysis of the regenerated catalyst exhibited comparable behavior to that of a fresh sample, confirming the successful removal of organic matter.

The regenerated catalyst showed fully revived activity in sorbitol dehydration (Figure 11c). Conversion even improved slightly to 49% following regeneration (Table S7). Repeated regenerative reuse resulted in a small overall decline in activity but achieved at least 35% conversion across all five recycle/regeneration experiments with a similar distribution of products (Table S7). Additional trials of regenerative reuse exhibited similar consistency in selectivities across three cycles (Table S8).

Spectroscopic analyses of the spent catalysts indicate notable differences from fresh and regenerated samples. Infrared spectra obtained via DRIFTS (Figure S13) exhibit two new distinct regions corresponding to C–H bending (δ_C–H_) and stretching (ν_C–H_) modes from surface organic deposits, however absent in the regenerated sample. Moreover, XPS survey scans presented in Figure S14a reveal peaks characteristic of O, Zr, C, and S in all samples. High-resolution spectra of Zr 3d, C 1s, and S 2p (Figure S14b–d) provide a clearer insight into the changes to elemental surface makeup after reaction and regeneration. A marked increase in the intensity of the C 1s peak (Figure S14c) in the spent catalyst evinced the presence of organic deposits, also in good agreement with DRIFTS spectra in Figure S13. As shown in Table 4, the C atomic percentage (at. %) increased significantly after the reaction. Following regeneration treatment, the atomic S/Zr composition and C at. % were restored back to those in the fresh sample, suggesting that regeneration is not only effective in removing deposited organics built up from the reaction, but importantly that the recycling methodology and work-up limit sulfate leaching. By restricting surface sulfate losses, the productive life of the catalyst may be extended.

**Table 4:** XPS Elemental Surface Compositions of Fresh, Spent, and Regenerated Catalysts^a^

catalyst	S at. %	Zr at. %	C at. %	At. S/Zr
fresh	4.4	26	2.4	0.17
spent	3.0	22	13	0.14
regenerated	4.4	26	2.5	0.17

a Reaction conditions (spent): 150 °C, 5 min, 50 mg catalyst, 182 mg (1 mmol) sorbitol. Regeneration conditions: 500 °C, 5 h, flowing air. Atomic percentages (at%) obtained from S 2p, Zr 3d, O 1 s (not shown), and C 1 s.

## Conclusions

Sulfated zirconia (SZ) catalysts were prepared through a modified sol–gel procedure from zirconium propoxide and aqueous sulfuric acid with varying S/Zr ratio (0.1–1.0) and calcination temperature (425–625 °C). The described synthesis allowed for a systematic control over the acid site density (1.5–7.2 nm^–2^) as well as Brønsted/Lewis acid sites ratio (0.01–2.70) as determined by NH_3_-TPD and py-FTIR. The SZ catalysts were quite effective in the selective dehydration of sorbitol in the melt (no added solvent) at 150 °C with >70% yield of isosorbide (67% isolated yield). The reaction progress was followed by quantitative ^13^C NMR revealing 1,4-sorbitan as the major intermediate en route to the isosorbide product. Kinetic modeling of the time profiles was consistent with sequential cyclodehydration of sorbitol and five rate constants. The first step involved two competing dehydration pathways to 1,4-AHSO (*k*_1_) or the co-product 2,5-AHSO (*k*_3_). The ratio of *k*_1_/*k*_3_ determined the reaction selectivity. Subsequent cyclodehydration of 1,4-AHSO (*k*_2_) yielded the product isosorbide. Side reactions of the products to oligomeric humins (*k*_4_ and *k*_5_) were necessary in the modeling to account for the mass of total dissolved solids. The activation energies (*E_a_*) for *k*_1_ and *k*_2_ were determined to be around 90 kJ mol^–1^. While the selectivity as determined by the ratio of *k*_1_/*k*_3_ increases with temperature, so does the decomposition to oligomeric humins (*k*_4_ and *k*_5_). As a result, the optimal temperature was determined to be in the range 150–160 °C. The Brønsted acid surface density (eq 2) was found to be a descriptor for the rate of isosorbide formation, TOF_isosorbide_. The rate increases reaching a maximum at a density of ∼2.25 Brønsted sites·nm^–2^ and thereafter inverting at higher densities. Furthermore, a synergistic cooperation between adjacent Lewis and Brønsted acid sites was uncovered. While the Brønsted sites are needed to induce cyclic dehydration, a B/L ratio of ∼1.0 is necessary to direct the primary hydroxyl groups toward dehydration, leading to selective isosorbide formation. TGA, DRIFTS, and XPS analyses of the spent catalyst revealed that the SZs were deactivated primarily by organic matter deposition. Consequently, SZ catalysts were successfully regenerated by thermal treatment at 500 °C under air. The high activity and tunable acid properties of these recyclable, sol–gel sulfated zirconia catalysts indicate good promise in their potential extension to broader melt-phase processing of other biomass-derived oxygenates.

## Supporting Information Available

Sorbitol cyclodehydration mechanism, catalyst synthesis scheme, analytical data, kinetics, and characterization data (PDF)
