# Supplementary material for: Markedly Improved Catalytic Dehydration of Sorbitol to Isosorbide by Sol–Gel Sulfated Zirconia: A Quantitative Structure–Reactivity Study
Source: ACS Catal. 2023 Jul 19;13(15):10137–52. doi: 10.1021/acscatal.3c00755 (PMC10411504; doi:10.1021/acscatal.3c00755)
Supplement: Supplementary file 1 — cs3c00755_si_001.pdf [file cs3c00755_si_001.pdf]

Supporting Information

Markedly improved catalytic dehydration of sorbitol to isosorbide by sol-gel sulfated zirconia: a quantitative structure-reactivity study

Jack T. Hopper,[a] Ruining Ma,[a†] James B. Rawlings,[b] Peter C. Ford,[a*] Mahdi M. Abu-Omar[a,b*]

[a] Department of Chemistry and Biochemistry, [b] Department of Chemical Engineering, University of California Santa Barbara, Santa Barbara, California 93106, United States

[*]Corresponding author email address: mabuomar@ucsb.edu; pcford@ucsb.edu

Table of Contents

Schemes

Scheme S1. Sorbitol dehydration mechanism S3

Figures

Figure S1. ^{13}C NMR inversion recovery S4
Figure S2. Modified SZ sol-gel synthesis, ‘alkoxide-to-acid’ S5
Figure S3. HPLC analysis with q^{13}C NMR comparison S6
Figure S4. ^{13}C NMR time-resolved spectra with all detected reaction species labeled S7
Figure S5. Reaction temperature optimization of sorbitol dehydration over 0.5-SZ-625 .... S8
Figure S6. BET transform plot of NS S9
Figure S7. Pore size distributions S9
Figure S8. X-ray diffractograms S10
Figure S9. NH_3-TPD of 0.5-SZs S10
Figure S10. Py-FTIR of 0.5-SZs S11
Figure S11. TGA of uncalcined 0.5-SZ-625 .... S12
Figure S12. Sorbitol dehydration over limited quantity of H_2SO_4 with and without SZ S13
Figure S13. DRIFTS of fresh, spent, and regenerated 0.5-SZ-625 .... S14
Figure S14. XPS of fresh, spent, and regenerated 0.5-SZ-625 .... S15

Tables

Table S1. Sorbitol dehydration over commercial and SZ catalysts S16
Table S2. Total dissolved solids experiment S17
Table S3. 140 – 170 °C time profiles of 0.5-SZ-625 .... S18
Table S4. SZ physical properties S19
Table S5. Elemental analyses S20
Table S6. Acid characterization S21
Table S7. Recycling studies using 0.5-SZ-625 .... S22
Table S8. Recycling reproducibility of 0.5-SZ-625 .... S23

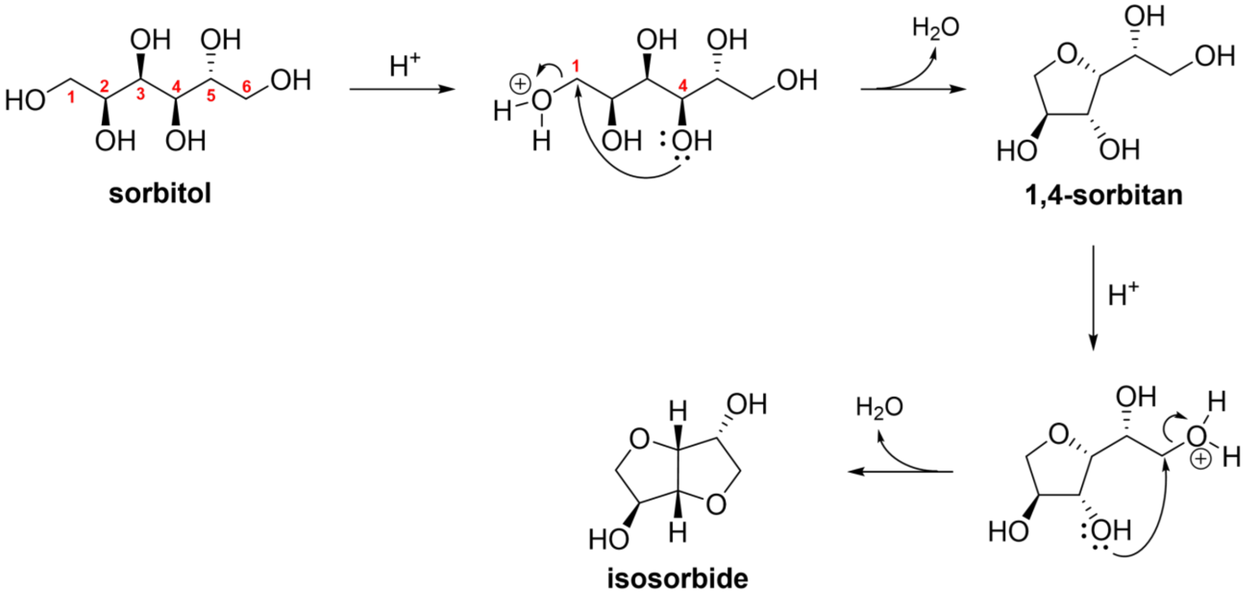

Scheme S1. Proposed mechanism of double cyclodehydration of sorbitol to isosorbide.

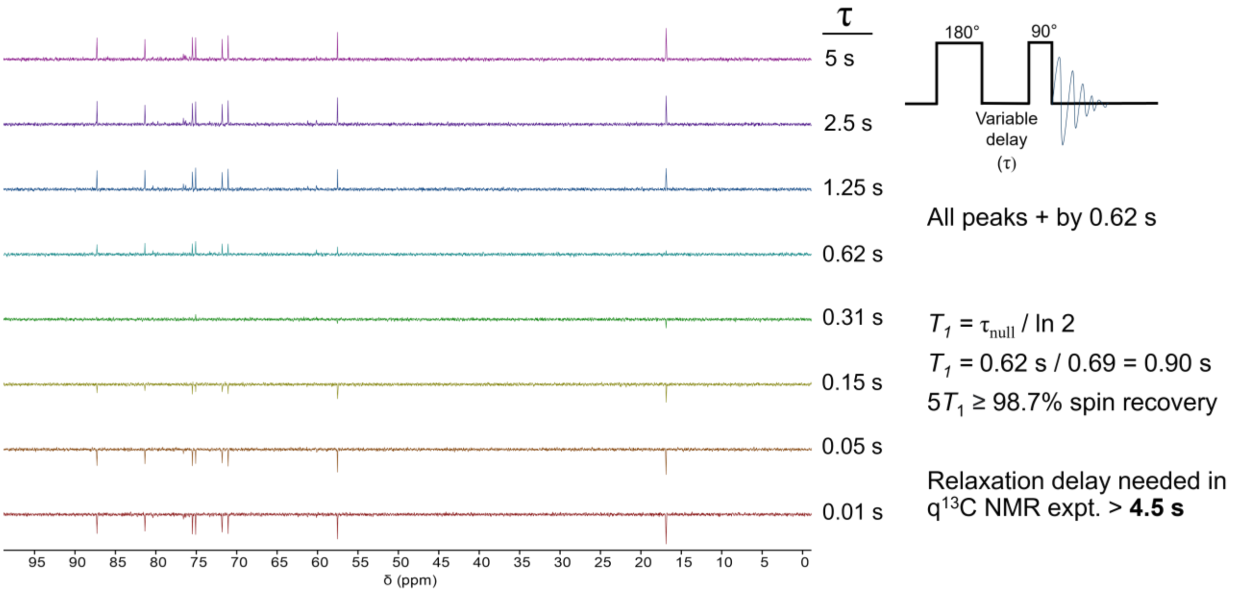

Figure S1. ^{13}C NMR inversion recovery for estimation of longest T_1 in reaction sample with 10 mM $GdCl_3$ after complete conversion containing isosorbide, 2,5-mannitan, 2,5-iditan, and ethanol (internal standard). 98.7% spin recovery at $t = 5T_1$ is obtained through simple exponential recovery, $(1 - 2e^{(-t/T_1)})$M, where M is the equilibrium population. Significant line broadening of sorbitol peaks was observed with $GdCl_3$ likely due to the formation of a metal chelate, preventing the use of $GdCl_3$ in samples with incomplete conversion. A relaxation delay of 8 s was ultimately used in analyses employing $GdCl_3$ to further improve confidence in integration through additional spin recovery time.

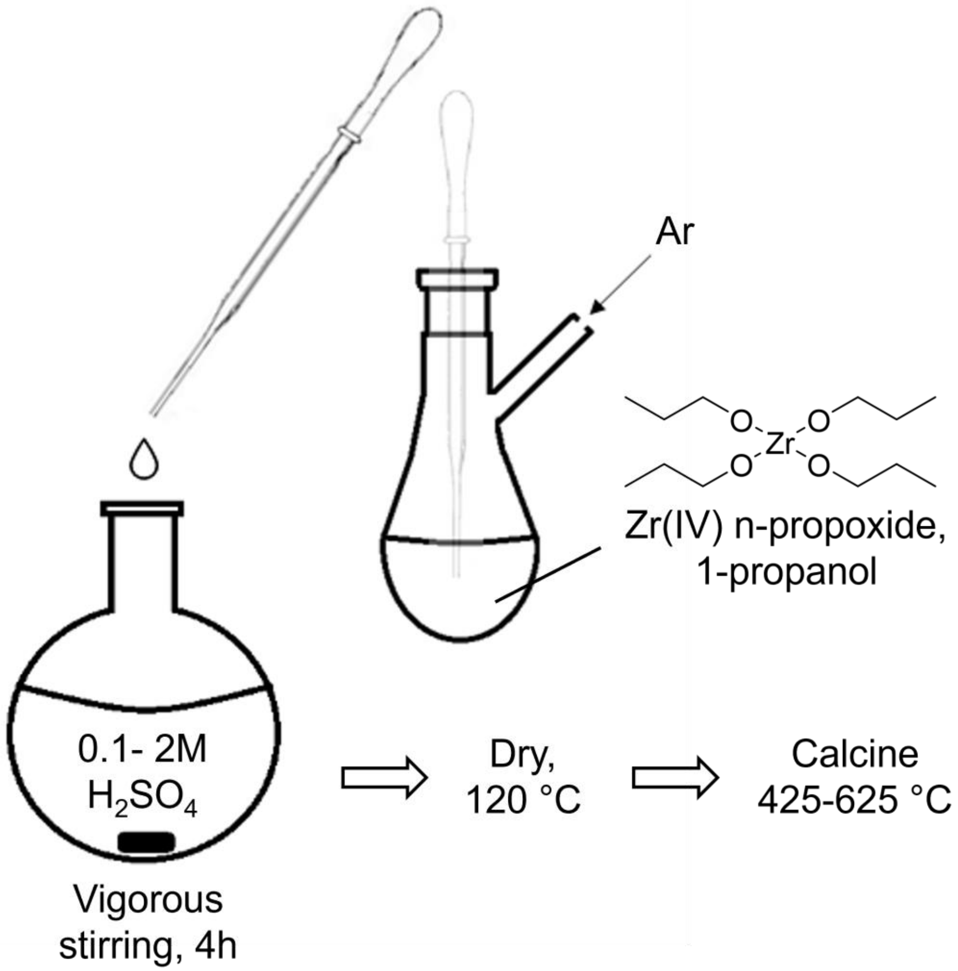

Figure S2. Modified SZ sol-gel synthesis, 'alkoxide-to-acid'.

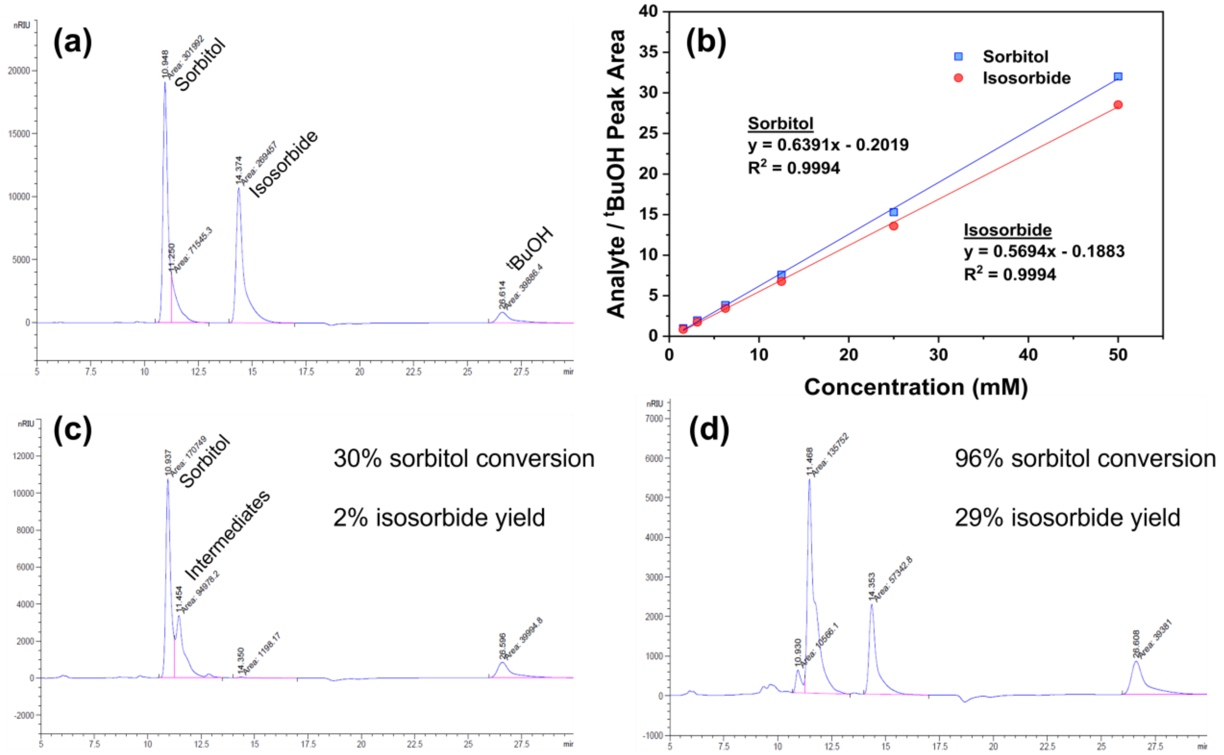

Figure S3. (a) HPLC (high-performance liquid chromatography) chromatogram of solution containing 12.5 mM sorbitol and isosorbide with 5 mM *tert*-butanol (^tBuOH) as internal standard. (b) The resulting calibration curves were used for quantification via HPLC of (c) 3 min and (d) 30 min reactions over SZ. To provide comparison to HPLC, quantitative ^{13}C NMR was used on the same reaction mixtures. Sorbitol conversion / isosorbide yield from NMR were determined to be 26% / 1% and 99% / 31% from 3 min and 30 min reactions, respectively. All analyses were performed on an Agilent 1260 Infinity Quaternary HPLC system equipped with an Agilent Hi-Plex H column (300 × 7.7 mm, 70 °C) and refractive index detector (RID, G1362A). 5 mM H_2SO_4 was used as the mobile phase with a flow rate of 0.5 mL/min. Integration of the sorbitol peak in standard solutions was adjusted to the location of the local minimum between sorbitol and anhydrohexitol intermediate peaks in reaction solutions due to overlap. Reaction conditions: 150 °C, 25 mg 0.5-SZ-625 catalyst, 91 mg (0.5 mmol) sorbitol.

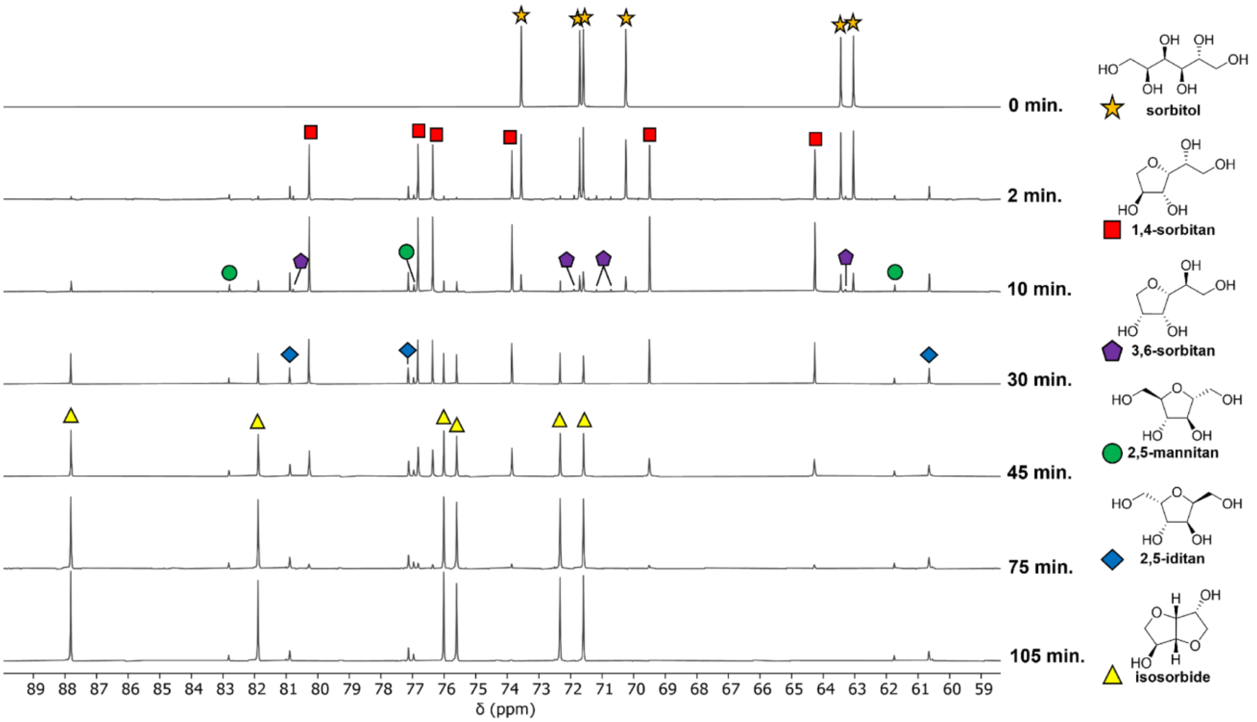

Figure S4. ^{13}C NMR time-resolved spectra with the detected reaction species labeled. Remaining 3,6-sorbitan peak is located at 71.69 ppm, overlapped by C4 of sorbitol (71.71 ppm). Reaction conditions: 150 °C, 25 mg 0.5-SZ-625 catalyst, 91 mg (0.5 mmol) sorbitol.

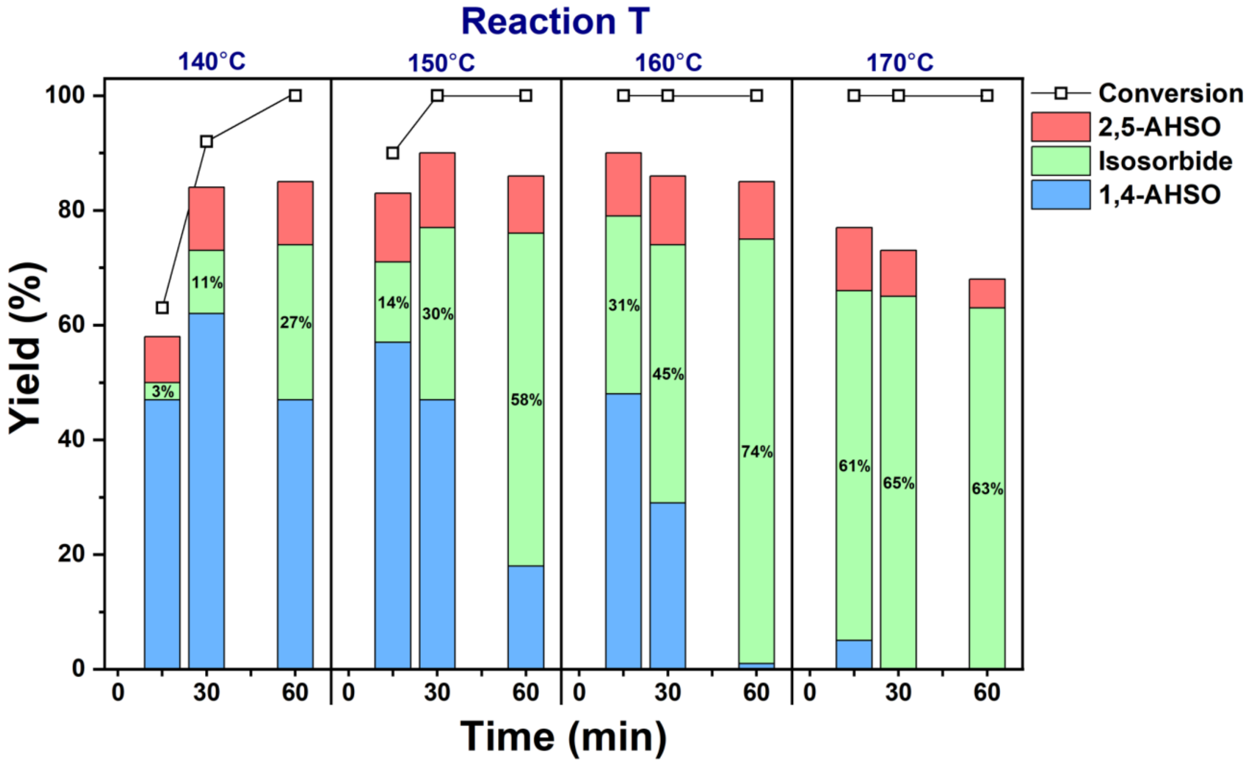

Figure S5. Reaction temperature optimization of sorbitol dehydration. Remaining mass is attributed to humins is recovered as part of total dissolved solids analysis. Data shown is abbreviated and presented for illustrative purposes. Full reaction data available in Table S3. 1,4-AHSO = 1,4-sorbitan and 3,6-sorbitan; 2,5-AHSO = 2,5-mannitan and 2,5-iditan. Reaction conditions: 25 mg 0.5-SZ-625, 0.25-1 h, 91 mg (0.5 mmol) sorbitol. Conversion and yields calculated by ^{13}C NMR using ethanol as an internal standard.

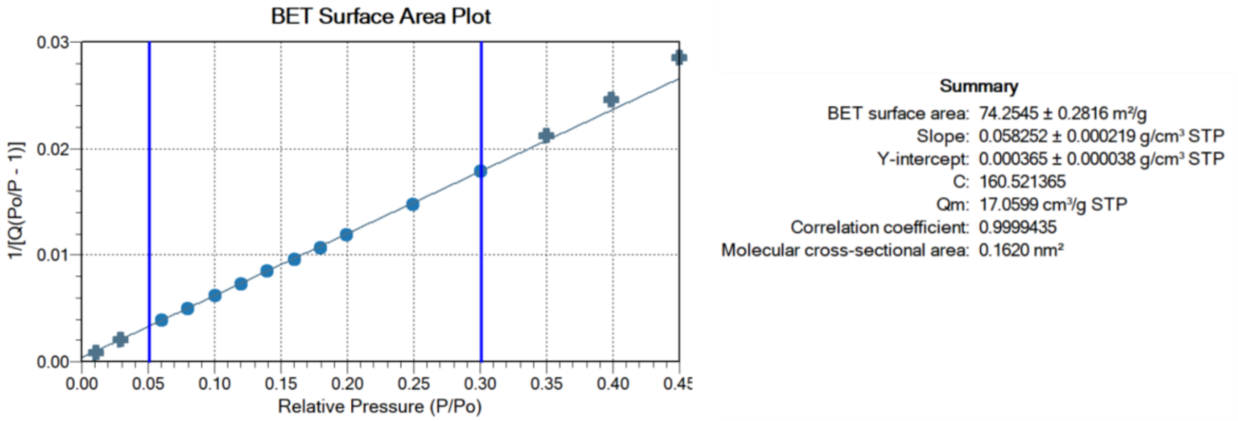

Figure S6. BET transform plot in Micromeritics Flex software of NS-625 catalyst. The relative pressure range of 0.05-0.3 was used in determination of BET equation parameters for calculation of surface area.[1]

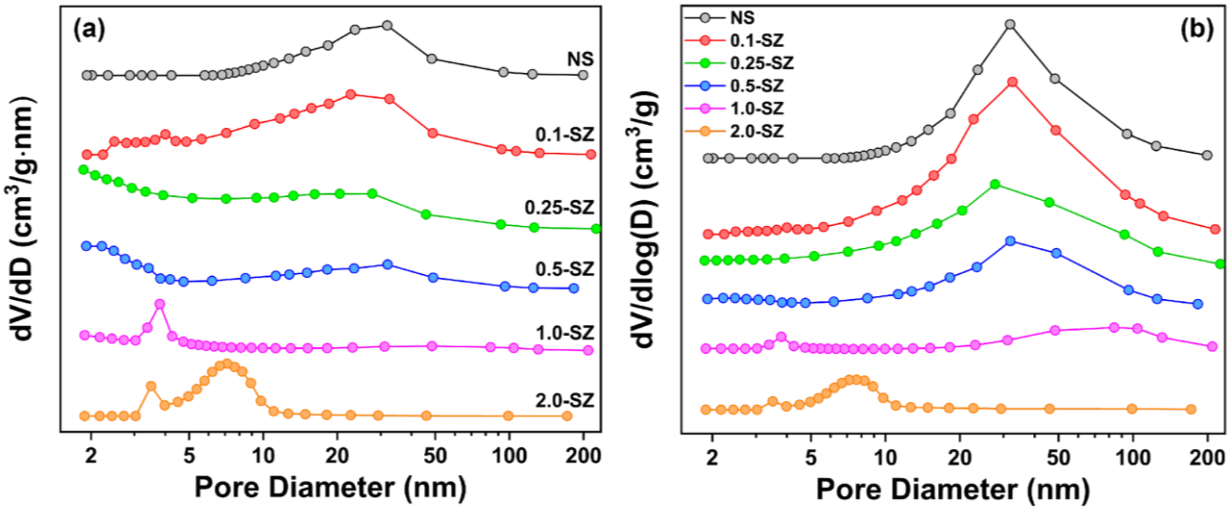

Figure S7. (a) Differential and (b) log differential pore size distributions of catalysts calcined at 625 °C. Data obtained from applying BJH model to N_2 desorption isotherm.

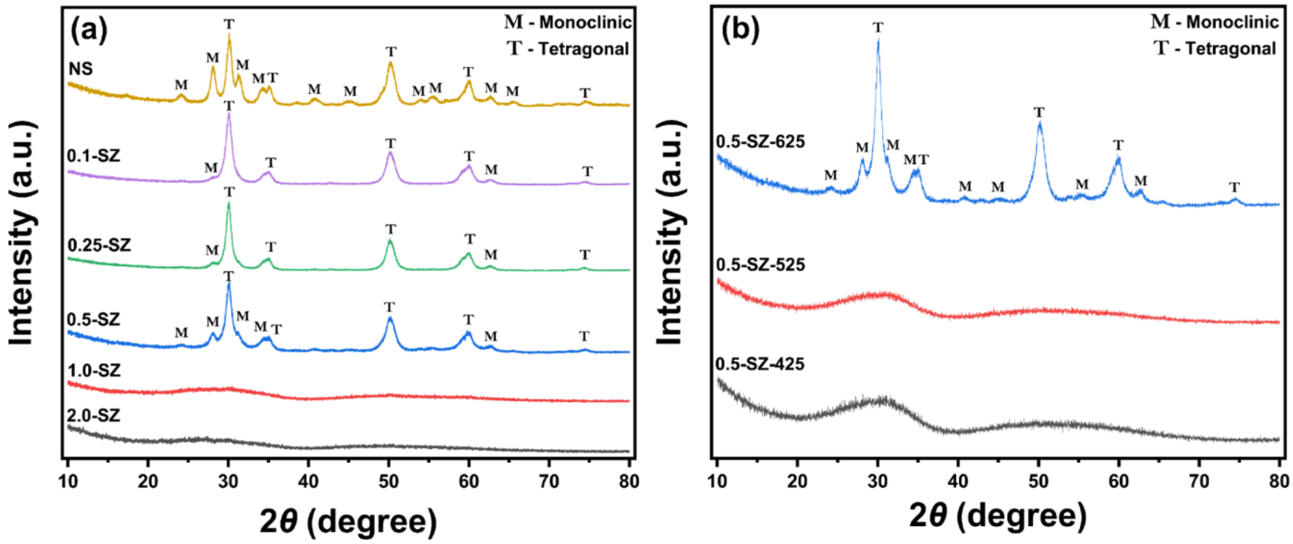

Figure S8. XRD diffractograms of (a) catalysts calcined at 625 °C with different S/Zr ratios and (b) 0.5-SZ catalysts calcined at different temperatures (425, 525, and 625 °C). Spectra were recorded on a PANlytical Empyrean powder diffractometer (Cu K_{α} source) in the range of 2θ = 10 – 80°.

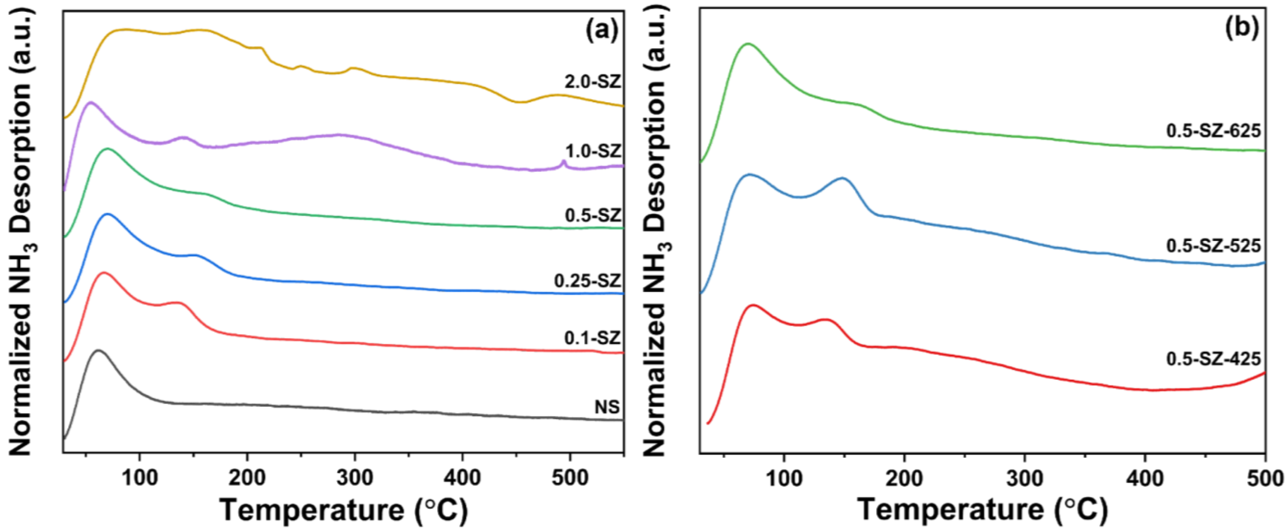

Figure S9. NH_3-TPD of (a) SZ catalysts calcined at 625 °C with varying S/Zr and (b) 0.5-SZ catalysts calcined at 425, 525, or 625 °C. Desorption spectra from heating (10 °C/min) were collected using an Autochem II 2920 chemisorption analyzer following dehydration (150 °C / 1 h / He) and exposure to NH_3 (30 °C / 0.5 h / 10% NH_3 in He).

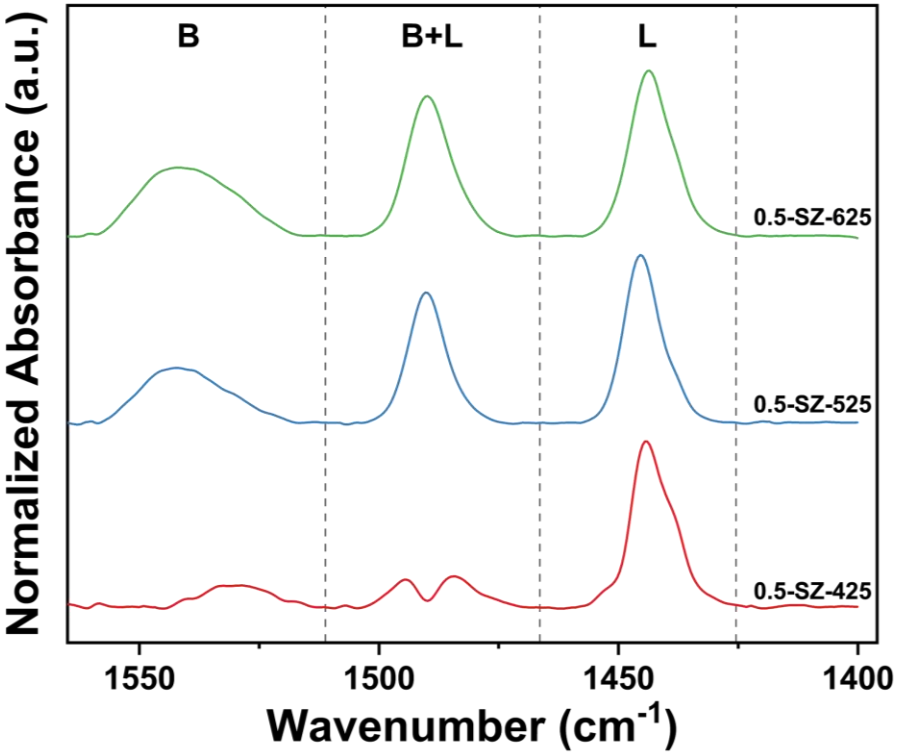

Figure S10. FTIR spectra of 0.5-SZ catalysts with varying calcination temperatures after dehydration (150 °C / 1 h / Ar) and subsequent pyridine adsorption (30 °C / 0.5 h / bubbling Ar). A Thermo Scientific Nicolet iS10 FTIR spectrometer was utilized for detection of pyridine coordinated to Brønsted (**B**, 1530 – 1540 cm^{-1}) or Lewis acid sites (**L**, 1445 cm^{-1}).[2]

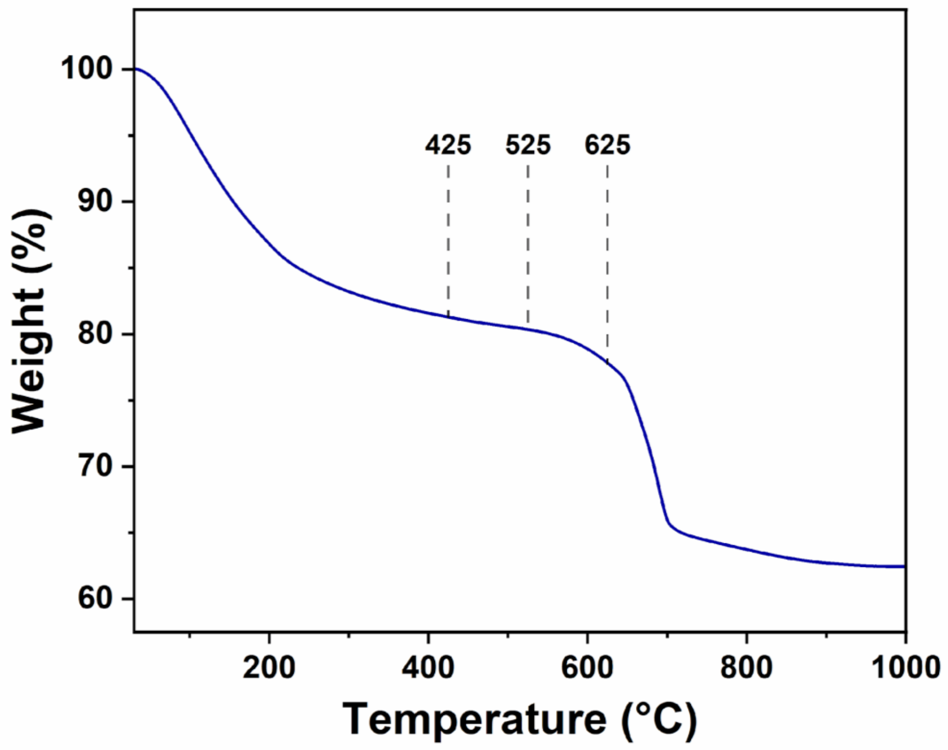

Figure S11. Thermogravimetric analysis of uncalcined 0.5-SZ precatalyst. Thermal behavior was recorded using a Discovery TGA 5500 instrument, ramping at a rate of 10 °C/min. The three calcination temperatures used in SZ syntheses are labeled with dashed lines.

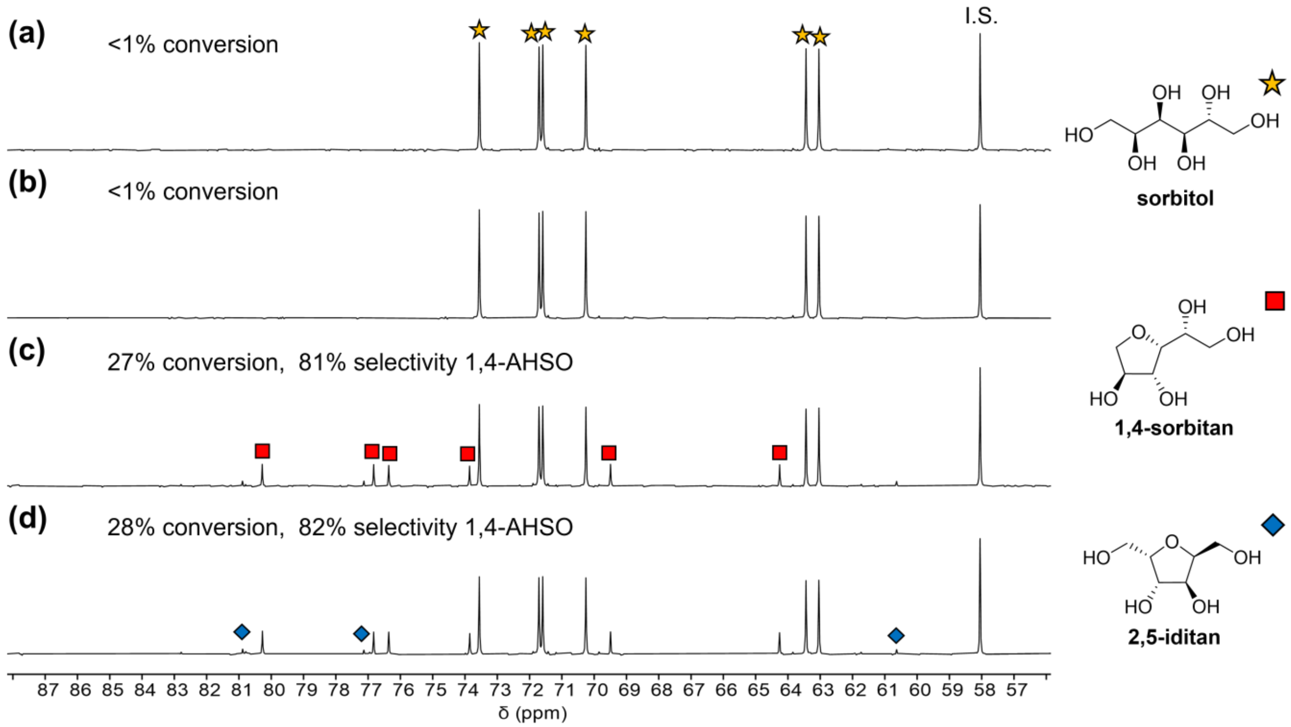

Figure S12. Quantitative ^{13}C NMR spectra after reaction of sorbitol without SZ with (a) 0.1 mol% S as H_2SO_4 and (b) 1 mol% S as H_2SO_4 obtained from S elemental analysis of 0.5-SZ-625. Additional reactions were then conducted in the presence of SZ using identical volumes of (c) water to serve as control or (d) 1 mol% S as H_2SO_4. Conversion and yields calculated by using ethanol as an internal standard (I.S.). Reaction conditions (without SZ): 150 °C, 5 min, 170 µL *aq*. H_2SO_4, 91 mg (0.5 mmol) sorbitol. Reaction conditions (with SZ): 150 °C, 5 min, 25 mg 0.5-SZ-625 catalyst, 170 µL water or *aq*. H_2SO_4, 91 mg (0.5 mmol) sorbitol. Major species labeled. 3,6-sorbitan and 2,5-mannitan also detected as minor products.

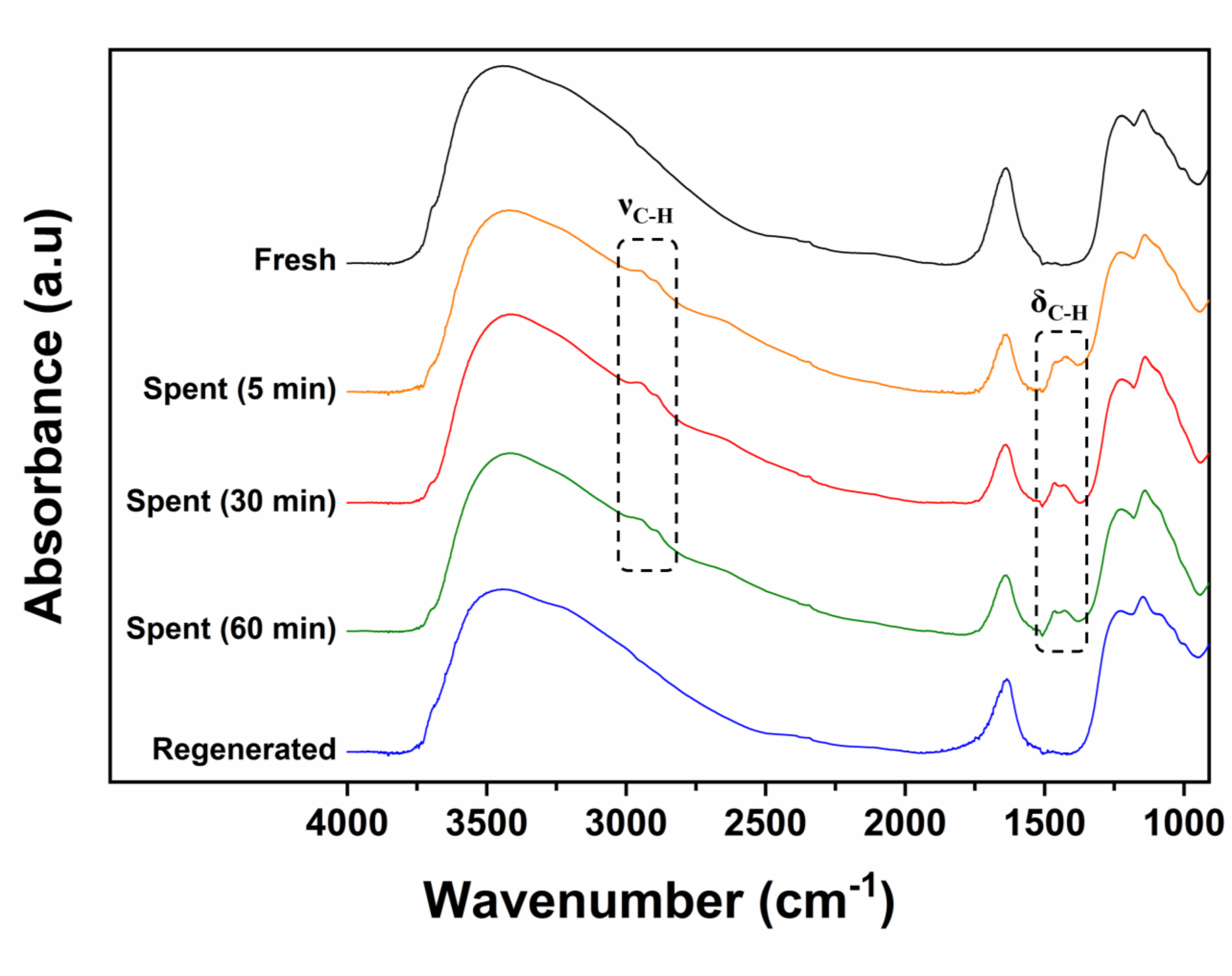

Figure S13. DRIFTS spectra of fresh, spent, and regenerated 0.5-SZ-625 catalysts recorded using a Thermo Scientific Nicolet iS10 FTIR spectrometer. Reaction conditions (spent): 150 °C, 50 mg catalyst, 182 mg (1 mmol) sorbitol. Regeneration conditions: 500 °C, 5 h, flowing air.

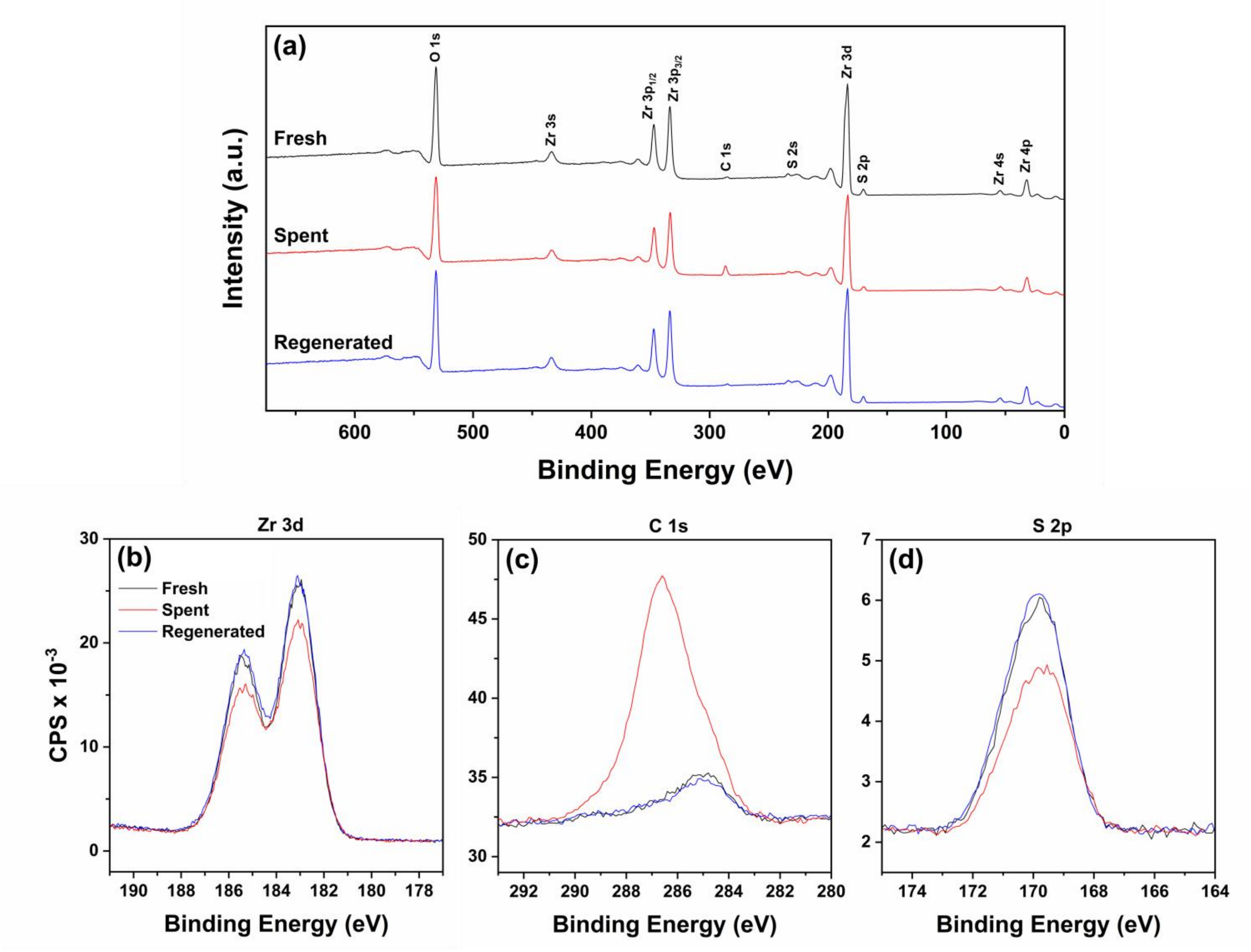

Figure S14. XPS (a) survey scans and (b) high-resolution spectra of Zr 3d, (c) C 1s, and (d) S 2p regions in fresh, spent, and regenerated 0.5-SZ-625 catalysts. Reaction conditions (spent): 150 °C, 5 min, 50 mg catalyst, 182 mg (1 mmol) sorbitol. Regeneration conditions: 500 °C, 5 h, flowing air.

Table S1. Sorbitol dehydration over SZ catalyst and other catalysts[a]

Catalyst	Sorbitol Conversion[b] (%)	Yield[b] (%)					
		1,4-sorbitan	3,6-sorbitan	2,5-iditan	2,5-mannitan	Isosorbide	Humins/Others[c]
None	0	0	0	0	0	0	0
Zeolite Na-Y	7	0	0	0	0	0	7
ZSM-5	16	4	4	1	0	4	3
ZrO_2	13	0	0	0	0	0	13
NS-625	13	0	0	0	0	0	13
0.1-SZ-425	48	31	2	5	2	2	6
0.1-SZ-525	>99	1	0	4	2	64	29
0.1-SZ-625	>99	2	0	6	3	63	26
0.25-SZ-425	>99	17	0	7	3	48	25
0.25-SZ-525	>99	4	0	7	3	67	19
0.25-SZ-625	>99	0	0	4	2	69	25
0.5-SZ-425	>99	11	0	8	3	64	14
0.5-SZ-525	>99	10	0	7	3	63	16
0.5-SZ-625	>99	0	0	5	3	74	18
1.0-SZ-425	>99	5	0	8	3	64	20
1.0-SZ-525	>99	34	0	8	4	36	18
1.0-SZ-625	>99	39	0	9	5	34	13
2.0-SZ-425	>99	0	0	0	0	35	65
2.0-SZ-525	>99	0	0	0	0	33	67
2.0-SZ-625	>99	0	0	0	0	24	76

[a]Reaction conditions: 150 °C, 2 h, 25 mg catalyst, 91 mg (0.5 mmol) sorbitol. [b]Calculated by ^{13}C NMR using ethanol as an internal standard. [c]From total dissolved solids experiment, see Table S2.

Table S2. Total dissolved solids (TDS) experiment.[a]

**Pre-reaction**	Sorbitol	91.1 mg	
	Catalyst	25.1 mg	
	**Total**	**116.2 mg**	
**NMR Quantification**	Dry, Post-Reaction Material[b]	73.0 mg	
		Yield[c]	Mass
	Isosorbide (146.14 g/mol)	64%, 0.32 mmol	46.8 mg
	1,4-AHSO (164.16 g/mol)	0%, 0 mmol	0 mg
	2,5-AHSO (164.16 g/mol)	6%, 0.03 mmol	4.9 mg
	Mass Balance from NMR	0.35/0.5 mmol = 70%	
	Remaining Post-Reaction Mass	73.0 – 46.8 – 4.9 = **21.3 mg**	
**Post-reaction**	Catalyst (+ coke)	29.0 mg	
	Isosorbide	46.8 mg (0.32 mmol)	
	2 mol H_2O evolved/mol isosorbide	11.5 mg (0.64 mmol)	
	2,5-AHSO	4.9 mg (0.03 mmol)	
	1 mol H_2O evolved/mol isosorbide	0.5 mg (0.03 mmol)	
	Humins[d]	21.3 mg	
	H_2O evolved from humins formation[e]	2.7 mg (0.15 mmol)	
	**Total**	**116.7 mg, 100.4%**	

[a]Reaction conditions: 150 °C, 2 h, 25 mg 0.1-SZ-525 catalyst, 91 mg (0.5 mmol) sorbitol. [b]After the allotted time, the catalyst was separated from the reaction mixture using a filter. The post-reaction material and catalyst were allowed to dry under vacuum for 48 h before masses were taken. [c]Calculated by ^{13}C NMR using ethanol as an internal standard. [d]Assuming remaining post-reaction mass not accounted for in NMR are humins. [e]Primary mode of humins formation is due to intermolecular condensation reactions,[3] such as etherification, thereby generating one molecule of water per humin monomer subunit.

Table S3. Temporal appearance and/or disappearance of substrate, intermediates, product, and byproducts as a function of temperature over 0.5-SZ-625.[a]

Temperature	Time (h)	Conversion[b] (%)	Yield[b] (%)		
			1,4-AHSO	2,5-AHSO	Isosorbide
**140 °C**	0.25	63	47	8	3
	0.5	92	62	11	11
	1	>99	47	11	27
	2	>99	7	10	66
	3	>99	0	8	71
**150 °C**	0.033	44	37	5	2
	0.083	59	44	7	4
	0.167	82	59	10	7
	0.25	90	57	12	14
	0.5	>99	47	13	30
	0.75	>99	32	12	43
	0.917	>99	24	11	52
	1	>99	18	10	58
	1.25	>99	7	10	68
	1.75	>99	3	10	72
	2	>99	0	9	76
	3	>99	0	8	74
**160 °C**	0.033	50	38	6	2
	0.167	89	61	10	10
	0.25	>99	48	11	31
	0.5	>99	29	12	45
	1	>99	1	10	74
	2	>99	0	6	70
	3	>99	0	4	64
**170 °C**	0.033	79	59	12	7
	0.083	88	58	13	10
	0.167	>99	51	14	23
	0.25	>99	5	11	61
	0.5	>99	0	8	65
	1	>99	0	5	63
	2	>99	0	2	55
	3	>99	0	1	52

[a]Reaction conditions: 25 mg 0.5-SZ-625 catalyst, 91 mg (0.5 mmol) sorbitol. [b]Calculated by ^{13}C NMR using ethanol as an internal standard.

Table S4. Structural and physical properties of all SZ catalysts with varying H_2SO_4 concentration and calcination temperature.

Catalyst	S_{BET}[a] (m^2/g)	Pore Volume[b] V_p (mL/g)	Pore Diameter[c] D_p (nm)	Tetragonal Phase[d] (%)
NS-625	75	0.600	31.2	52
0.1-SZ-425	213	0.752	16.5	Amorphous
0.1-SZ-525	176	0.840	19.1	100
0.1-SZ-625	133	0.846	23.8	83
0.25-SZ-425	143	0.623	21.2	Amorphous
0.25-SZ-525	136	0.666	23.5	Amorphous
0.25-SZ-625	127	0.689	21.3	81
Uncalcined 0.5-SZ	127	0.657	22.6	–
0.5-SZ-425	88	0.498	26.7	Amorphous
0.5-SZ-525	83	0.497	26.5	Amorphous
0.5-SZ-625	81	0.373	18.6	69
1.0-SZ-425	56	0.268	26.2	Amorphous
1.0-SZ-525	49	0.219	22.4	Amorphous
1.0-SZ-625	44	0.158	18.3	Amorphous
2.0-SZ-625	29	0.077	7.5	Amorphous

[a]Surface area (S_{BET}) determined from BET analysis on adsorption branch. [b]Calculated from BJH analysis on desorption branch. [c]Average mesopore diameter D_p determined from $D_p = 4V_p/S_{BET}$. The percentage of tetragonal phase was determined from the ratio of the integrated intensities of diffraction angles corresponding to tetragonal ($2\theta = 30.2°$) and monoclinic ($2\theta = 28.2°$ and 31.4°) zirconia, as described elsewhere.[4]

Table S5. Elemental analyses of SZ catalysts.

Catalyst	S content (wt%)	H content (wt%)
NS-625	0	0.31
0.1-SZ-625	1.48	0.18
0.25-SZ-625	1.72	0.25
0.5-SZ-Uncalcined	8.07	1.68
0.5-SZ-425	9.19	0.53
0.5-SZ-525	8.76	0.39
0.5-SZ-625	2.18	0.46
1.0-SZ-625	12.98	0
2.0-SZ-625	13.58	0.94

Table S6. Acid characterization of SZ catalysts

Catalyst	Acid site loading[a] (μmol NH_3/g)	Bronsted/Lewis Ratio[b]	Bronsted acid loading (μmol NH_3/g)	Bronsted acid site density (nm^{-2})	Total acid site density (nm^{-2})
NS-625	228	0.01	2	0.02	1.83
0.1-SZ-425	652	0.30	152	0.43	1.84
0.1-SZ-525	431	1.36	248	0.85	1.48
0.1-SZ-625	371	1.04	189	0.86	1.68
0.25-SZ-425	751	0.21	128	0.54	3.16
0.25-SZ-525	683	0.33	171	0.76	3.02
0.25-SZ-625	420	0.73	177	0.84	1.99
0.5-SZ-425	470	0.31	110	0.76	3.21
0.5-SZ-525	428	0.93	206	1.49	3.10
0.5-SZ-625	510	1.16	274	2.04	3.79
1.0-SZ-425	543	2.70	396	4.26	5.83
1.0-SZ-525	589	0.78	258	3.17	7.24
1.0-SZ-625	132	0.41	38	0.52	1.81

[a]NH_3-TPD analysis. [b]FTIR analysis of adsorbed pyridine.

Table S7. Recycling tests over 0.5-SZ-625 with and without regeneration[a]

Catalyst Use	Sorbitol Conversion[b] (%)	Yield[b] (%)					
		1,4-sorbitan	3,6-sorbitan	2,5-iditan	2,5-mannitan	Isosorbide	Humins/Others
1st	43	31	1	4	2	2	3
2nd	9	4	1	1	0	0	3
3rd[c]	49	36	2	5	2	2	2
4th[c]	41	29	2	3	2	2	3
5th[c]	38	27	2	3	1	1	4
6th[c]	39	29	2	3	1	1	3
7th[c]	35	24	2	3	1	1	4

[a]Reaction conditions: 150 °C, 5 min, 50 mg 0.5-SZ-625 catalyst, 1 mmol (182 mg) sorbitol. [b]A small loss in catalyst mass occurred with each use following separation. Subsequent trials were scaled down to maintain the initial catalyst/substrate ratio. Conversion and selectivity calculated by ^{13}C NMR using ethanol as an internal standard. [c]Regeneration conditions: 500 °C, 5 h, under flowing air.

Table S8. Reproducibility tests of regenerative reuse using 0.5-SZ-625.

		Sorbitol Conversion[b] (%)	Selectivity[b] (%)		
			1,4-AHSO	2,5-AHSO	Isosorbide
**Trial 1**	1st Use	43	74	14	5
	2nd Use[c]	49	78	14	4
	3rd Use[c]	41	76	12	5
**Trial 2**	1st Use	44	73	14	5
	2nd Use[c]	50	78	14	4
	3rd Use[c]	55	74	13	5
**Trial 3**	1st Use	41	73	15	3
	2nd Use[c]	52	79	13	4
	3rd Use[c]	58	74	14	5

[a]Reaction conditions: 150 °C, 5 min, 50 mg 0.5-SZ-625 catalyst, 1 mmol (182 mg) sorbitol. A small loss in catalyst mass occurred with each use following separation. Subsequent trials were scaled down to maintain the initial catalyst/substrate ratio. [b]Conversion and selectivity calculated by ^{13}C NMR using ethanol as an internal standard. [c]Regeneration conditions: 500 °C, 5 h, under flowing air.

References

(1) Brunauer, S.; Emmett, P. H.; Teller, E. Adsorption of Gases in Multimolecular Layers. *J. Am. Chem. Soc.* **1938**, *60* (2), 309–319.

(2) Morterra, C.; Cerrato, G.; Bolis, V.; Di Ciero, S.; Signoretto, M. On the Strength of Lewis- and Brønsted-Acid Sites at the Surface of Sulfated Zirconia Catalysts. *J. Chem. Soc. Faraday Trans.* **1997**, *93* (6), 1179–1184.

(3) Liu, S.; Zhu, Y.; Liao, Y.; Wang, H.; Liu, Q.; Ma, L.; Wang, C. Advances in understanding the humins: Formation, prevention, and application. *Applications in Energy and Combustion Science*. **2022**, *10*, 100062.

(4) Ahmed, A. I.; El-Hakam, S. A.; Samra, S. E.; EL-Khouly, A. A.; Khder, A. S. Structural Characterization of Sulfated Zirconia and Their Catalytic Activity in Dehydration of Ethanol. *Colloids Surf. Physicochem. Eng. Asp.* **2008**, *317* (1–3), 62–70.
